# LipidFrag: Improving reliability of *in silico* fragmentation of lipids and application to the *Caenorhabditis elegans* lipidome

**DOI:** 10.1371/journal.pone.0172311

**Published:** 2017-03-09

**Authors:** Michael Witting, Christoph Ruttkies, Steffen Neumann, Philippe Schmitt-Kopplin

**Affiliations:** 1 Research Unit Analytical BioGeoChemistry, Helmholtz Zentrum München, German Research Center for Environmental Health, Ingolstaedter Landstrasse, Neuherberg, Germany; 2 Chair of Analytical Food Chemistry, Technische Universität München, Alte Akademie 10, D-85354 Freising-Weihenstephan, Germany; 3 Leibniz Institute of Plant Biochemistry, IPB Halle, Department of Stress and Developmental Biology, Weinberg, Halle, Germany; Karl-Franzens-Universitat Graz, AUSTRIA

## Abstract

Lipid identification is a major bottleneck in high-throughput lipidomics studies. However, tools for the analysis of lipid tandem MS spectra are rather limited. While the comparison against spectra in reference libraries is one of the preferred methods, these libraries are far from being complete. In order to improve identification rates, the *in silico* fragmentation tool MetFrag was combined with Lipid Maps and lipid-class specific classifiers which calculate probabilities for lipid class assignments. The resulting LipidFrag workflow was trained and evaluated on different commercially available lipid standard materials, measured with data dependent UPLC-Q-ToF-MS/MS acquisition. The automatic analysis was compared against manual MS/MS spectra interpretation. With the lipid class specific models, identification of the true positives was improved especially for cases where candidate lipids from different lipid classes had similar MetFrag scores by removing up to 56% of false positive results. This LipidFrag approach was then applied to MS/MS spectra of lipid extracts of the nematode *Caenorhabditis elegans*. Fragments explained by LipidFrag match known fragmentation pathways, e.g., neutral losses of lipid headgroups and fatty acid side chain fragments. Based on prediction models trained on standard lipid materials, high probabilities for correct annotations were achieved, which makes LipidFrag a good choice for automated lipid data analysis and reliability testing of lipid identifications.

## Introduction

Metabolite and lipid identification represents the current bottleneck in metabolomics and lipidomics. The diversity of the lipidome is huge, with estimates of up to 100,000 different possible lipid structures. This is based on the combinatorial composition of different defined building blocks, which include fatty acids, long-chain bases, glycerol-phosphate, various head groups and many more [1]. Lipids fulfill several cellular functions, including storage of energy, building blocks of membranes, and signaling.

Several efforts have been made to catalog lipid diversity. Lipidat was one of the first electronic lipid databases [2], and contained 11,000 records, LIPIDBANK (initiated in 1989) contains just over 7,000 records as of 2013, and can still be browsed on the (http://lipidbank.jp/) [3].

The Lipid Maps database and classification system structure database (LMSD) [4] is a widely used resource for a systematic classification of lipids. It divides lipids into the eight major classes: fatty acyls (FA), glycerolipids (GL), glycerophospholipids (GP), sphingolipids (SP), sterol lipids (ST), prenol lipids (PR) saccharolipids (SL) and polyketides (PK), each with several subclasses. Lipid Maps contains currently 40,360 structures (accession date 4/2/15) and is accessible via the web (www.lipidmaps.org). LipidHome was developed at the European Bioinformatics Institute (EBI) and is a database of theoretical lipids with 20,297 species and 36 million theoretical sub species [5]. SwissLipids as another resource contains 244,000 known and theoretically lipids [6].

Multiple lipids have similar physicochemical properties which complicates their analysis. Nowadays, two different types of lipid analyses are commonly performed: Liquid chromatography-mass spectrometry (LC-MS) based lipidomics, or shotgun lipidomics. The latter uses direct infusion of the raw lipid extract into a mass spectrometer (MS) and acquisition of multi-stage mass spectra for precise quantification of lipid species using low and high resolution MS [7–10]. High resolution MS, MS/MS and ITMS^3^ have been employed for structural characterization and quantification of lipids from mouse cerebellum and hippocampus [10]. In contrast to shotgun lipidomics, LC-MS based lipidomics uses chromatographic separation of lipid species followed by mass spectrometric detection, which allows differentiation of isomeric lipid species. Common lipid profiling methods use C8 or C18 reversed phase columns and an acetonitrile/isopropyl alcohol (ACN/iPrOH) gradient. This method allows detection of phospho- and glycerolipids as well as other lipids in a single run [11, 12] and is typically coupled to high-resolution accurate mass Q-ToF or Orbitrap instruments for non-targeted profiling of as many lipids as possible. The high mass resolution and accuracy helps to annotate MS features with known lipids from different databases or to calculate molecular formulas of possible lipids species. However, several lipids, even from different lipid classes, can have identical molecular formulas, e.g. phosphatidylcholine (PC) and/or phosphatidylethanolamine (PE) species, such that a definite identification is impossible from the mass and even molecular formula alone. Searching LipidMaps for molecules having the same molecular formula or exact mass can result in up to 115 candidates with one single molecular formula. Taking into account possible adducts during the ionization process the number increases further. For example the sodium adduct ([M+Na]^+^) of PC(18:0/20:1) and the [M+H]^+^ adduct of PC(18:0/22:4) have a mass difference of 0.0024, which reflects a deviation of 2 ppm. A more extreme example is the formic acid adduct of PC(16:0/20:1) [M+HCOO]^-^ and the [M-H]^-^ of PS(17:0/22:0) having exactly the same molecular formula. Today’s ultrahigh resolution mass spectrometer, like Orbitrap or FT-ICR-MS instruments can reach mass errors below 1 ppm, but even with these ultrahigh resolution MS instruments it is only possible to accurately calculate a molecular formula, whereas no information about the lipid class, structure or fatty acid side chain composition is available [13]. Thus, tandem mass spectrometry (MS/MS) is needed to provide further information for a more reliable annotation. Fragmentation in positive ion mode can help to reveal the lipid class by neutral losses of lipid head groups, whereas negative ion mode resolves the fatty acid composition and position [14]. Data dependent acquisition (DDA) offers the possibility to collect several hundred MS/MS spectra for identification of metabolites or lipids during chromatographic runs [15].

Interpretation of the resulting MS/MS spectra, especially in high-throughput studies, is rather limited and manual analysis of several hundreds to thousands of MS/MS spectra is not feasible. To speed up identification, comparison against reference spectral databases is possible, but the lipid coverage in these databases is sparse. Lipid Maps currently contains only few hundreds low resolution MS/MS spectra, while MassBank has 3,158 records on both low and high resolution instruments covering 707 unique lipids [16].

*In silico* fragmentation has been suggested as a possible solution to analyze MS/MS spectra without the need of reference spectral databases [17]. LipidBlast is a spectral library that includes a 212,516 *in silico* generated tandem mass spectra covering 119,200 compounds from 26 lipid classes [18]. More recently, Greazy, an approach for identification of phospholipids from MS/MS data was presented which includes the estimation of false discovery rates (FDR). The modul LipidLama, integrated in Greazy, uses kernel density estimation to fit non-parametrized models to distinguish false and true lipid assignments. The cutoff score for a putative correct lipid assignment can then be defined by using a pre-defined FDR of e.g. 5% [19].

**Fig 1:**
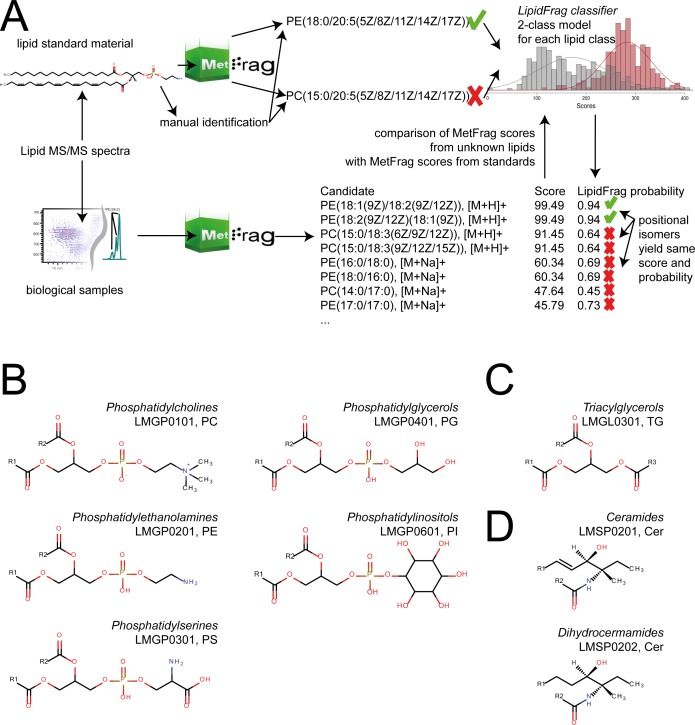
LipidFrag workflow and related lipid sub-classes. (A) Schematic drawing of LipidFrag workflow. MS/MS spectra from known lipids derived from lipid standard materials and from unknown lipids are subjected to MetFrag in silico fragmentation, whereby all possible precursor structures are taken into consideration. During training phase true positive identity and decoy candidates are used to calculate a 2-class classifier by which reliable results from unknown lipids can be identified. (B) Structures of detected phospholipid classes, phosphatidylethanolamines (PE, LMGP0201), phosphatidylcholines (PC, LMGP0101), phosphatidylglycerols (PG, LMGP0401), phosphatidylserines (PS, LMGP0301) and phosphatidylinositols (PI, LMGP0601) (C) Structure of triacylglycerols (TG, LMGL0301) (D) Structure of ceramides (Cer, LMSP0201) and dihydroceramides (Cer, LMSP0202).

**Fig 2:**
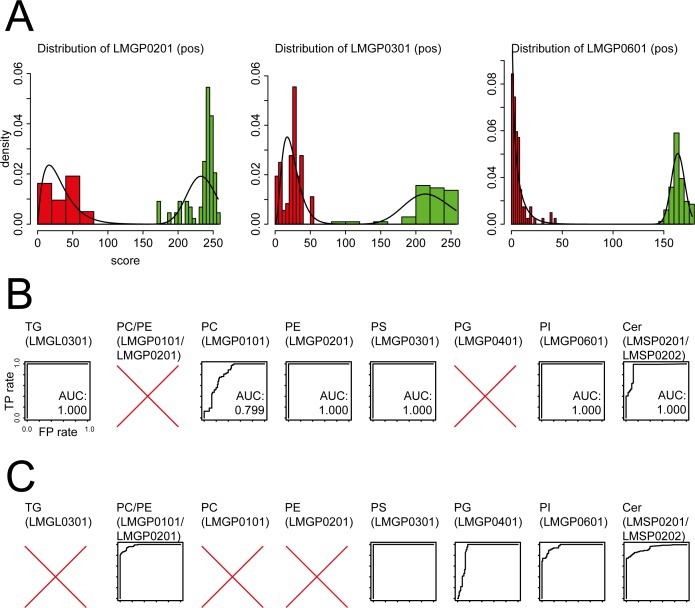
Visualization of input data and results obtained by LipidFrag. (A) Examples of histograms showing distribution of raw MetFrag score for the back- and foreground training dataset. (B) Receiver-Operator characteristics (ROC) derived from 10-fold cross-validation of MS/MS spectra from lipid standard materials detected negative ion mode. (C) Receiver-Operator characteristics (ROC) derived from 10-fold cross-validation of MS/MS spectra from lipid standard materials detected positive ion mode. In both panels, plots having no AUC value indicate that this lipid class was not detected in this ion mode concluding that there was not training data for classifiers available. All axes have the same scale.

In this study we present a workflow to improve the reliability of *in silico* MS/MS annotations of lipids. To achieve this, we introduce bayesian classifiers based on parametrised distributions and maximum-likelihood estimation to calculate a reliability score for a result to be a correct annotation among its lipid class, which is based on training data obtained from lipid standard materials and true positive manual identifications. This workflow consists of the annotation of precursor masses with possible lipid structures using MassTRIX [20–22], followed by MetFrag batch processing of candidates retrieved via the putative neutral masses derived from ion species annotation results. The performance was evaluated using MS/MS spectra obtained previously with UPLC-Q-ToF-MS/MS and data dependent acquisition (DDA) [23]. The lipid classes relevant for this paper are depicted in Fig 1, which included ceramides, different glycerophospholipids classes and glycolipids. Results from this training allowed the development of the central new feature in LipidFrag, the classifiers to predict the probability of a reliable MetFrag annotation for an unknown lipid class (Fig 2A). This is used to differentiate between good and poor identification results and to predict the underlying lipid main class of the precursor in high-throughput MS/MS experiments like in this case study performed with the lipid extract of *C*. *elegans*.

## Methods

### Chemicals

HPLC-grade methyl-tert-butyl ether (MTBE) and LC-MS-grade methanol (MeOH), iso-propanol (iPrOH), acetonitrile (ACN), ammonium formate and formic acid were obtained from Sigma-Aldrich. Water was purified using a Merck Millipore Integral water purification system with a resistance of 18 MΩ and TOC < 5 ppb.

### Lipid standard material preparation

Phosphatidylcholine preparation from chicken egg (840051P, Avanti Polar Lipids), Escherichia coli polar lipid extract (100600P, Avanti Polar Lipids), phosphatidyl serines from porcine brain (840032P, Avanti Polar Lipids), ceramide from porcine brain (860052P, Avanti Polar Lipids) and ceramide from chicken egg (860051P, Avanti Polar Lipids) were obtained from Avanti Polar Lipids (Otto Nordwald GmbH, Germany) and dissolved in MeOH at a concentration of 1 mg/mL. Additionally, L-alpha-Phosphatidylinositol sodium salt from Glycine max (P0639), Triglyceride mix (17811-AMP), 1,3-Dioleoyl-2-palmitioyl-glycerol (D1657), Glyceryl tritricosanoate (T1412), Glyceryl trioleate (T7140) and 1,2-Dilinoleoy-3-palmitoyl-rac-glycerol (D0301) were obtained from Sigma-Aldrich (Taufkirchen, Germany) and dissolved in either MeOH, MTBE, CHCl3 or solvent mixtures, depending on solubility. Different samples for analysis were prepared and diluted in ACN/iPrOH/water (65/30/5, v/v/v) to 10 μg/mL for analysis. The following lipid classes and standard samples were analysed and are named throughout the paper as indicated in brackets: Phophatidylcholines (PC, LMGP0101), phosphatidylethanolamines (PE, LMGP0201), phosphatidylglycerols (PG, LMGP04011), phosphatidylserines (PS, LMGP0301), phosphatidylinositols (PI, LMGP0601), ceramides (Cer, LMSP0201/ LMSP0202), and triacylglycerols (TG, LMGL0301).

### Lipid extraction from *C*. *elegans*

Lipids were extracted from young adult *C*. *elegans* using a modified method from Matyash et al. [24], described in [23]. The worms were washed off the plates and their metabolism was quenched with 500 μL -20°C MeOH. Samples were flash frozen in liquid nitrogen and stored at -80°C prior to extraction. Samples were then thawed on ice and 1.7 ml MTBE was added and samples were vortexed vigorously. *C*. *elegans* were lysed for 30 minutes in an ice cold ultrasonic bath, after which 420 μl of water was added and samples were sonicated for further 15 minutes. Phases were separated by centrifugation at 4°C and 14,000 rpm for 15 minutes. The upper organic phase was transferred to a 4 ml glass vial and the remaining lower phase was re-extracted with additional 650 μl MTBE for 15 minutes. After centrifugation the organic layers were combined and evaporated in a SpeedVac vacuum concentrator at 30°C for 0.5-1h. The residue was redissolved in 500 μl ACN/iPrOH/water (65/30/5, v/v/v).

### UPLC-Q-ToF-MS lipid profiling

Lipid profiling was performed as previously described [23]. Briefly, separation was achieved on a Waters Cortecs C18 column, 150mm x 2.1 mm ID, 1.6μm using a Waters Acquity UPLC (Waters, Eschborn, Germany) coupled to a Bruker maXis UHR-Q-ToF-MS (Bruker Daltonic, Bremen, Germany). Flow rate was 0.25 ml/min and column temperature was set to 50°C. Eluent A consisted of 60% ACN and 40% water, eluent B of 90% iPrOH and 10% ACN, both with 10 mM ammonium formate and 0.1% formic acid. Detection was carried out in positive and negative ion mode with data dependent acquisition with a scan rate of 5 Hz and selection of 2 precursors. Masses were excluded from DDA after 3 spectra and released from exclusion after 0.15 min. An absolute threshold of 1500 was used for selection.

### MS data processing

MS data was imported to Genedata Expressionist for Mass Spectrometry 8.2 (Genedata, Basel, Switzerland) for internal re-calibration, retention time alignment and peak picking. Files were exported to.xlsx format and further data handling was carried out in MS Excel. Lipids were annotated with a new in-house version of MassTRIX to also cover the adducts [M+NH4]^+^ and [M+HCOO]^-^, as well as [M+H]+, [M+Na]+ and [M-H]- and an absolute error of 0.005 Da [22].

MS/MS spectra were exported from the calibrated and aligned chromatograms from Genedata Expressionist for MS 8.2 as.mgf file. Only spectra associated with a detected feature were kept and converted to MetFrag batch files (available at http://msbi.ipb-halle.de/msbi/lipidfrag) using a custom Perl script. The neutral mass and formula for the batch file were obtained by annotation with MassTRIX, for all possible annotation results. Finally, spectra in batch files were de-isotoped using the CAMERA package with a custom R script [23]. The raw data in the open.mzML format and abundance matrix are available from the MetaboLights repository as MTBLS291 (http://www.ebi.ac.uk/metabolights/reviewerfec5e44e-fae6-46de-b55d-d2f22d425286).

### Manual lipid identification

Manual lipid identification was performed using known lipid fragmentation pathways. Information from both ionization modes was combined and matched via identical retention times, where available. For phospholipids, fragments used for identification included head group fragments and their respective neutral loss, loss fatty acid side chains and their carboxylate fragment. Position of fatty acids was inferred from intensity distributions of the respective [M-sn1], [M-sn2], sn1-fatty acid and sn2-fatty acid fragments. In the case of triacylglycerols neutral losses of fatty acid side chain as ammonium salt and the respective fragments were used. Ceramide species were identified based on typical sphingolipid fragments, e.g. loss of N-bound fatty acid and sphingoid base fragments.

Since exact position and stereochemistry of double bonds cannot be deduced from these experiments, all possible isomers were reported as potential identification for further processing with LipidFrag.

### LipidFrag identification

Batch query files were processed with the MetFrag command line tool (version 2.4 available at https://c-ruttkies.github.io/MetFrag/). Lipid Maps (LMSDFDownload18Mar14) was used as structure database. Candidates were considered within 20 ppm of the theoretical mass, and measured MS/MS peaks were matched against *in silico* fragments, generated with tree depth 3, with an error window of 0.01 Da + 15 ppm. The ion mode for the generated fragments were set according to the acquisition of the processed MS/MS peak list and the minimum peak intensity was set to 1000 arbitrary units. The resulting ranked candidate lists were filtered by the first part of the molecules’ InChIKey to eliminate stereo isomers and stored as CSV files, with the calculated MetFrag scores stored in the CSV columns. CSV files for MS/MS peak lists containing less than two informative MS/MS peaks were excluded from the evaluation. The score calculated by MetFrag was used to rank the known candidates of the standard spectra. Here, we always used the pessimistic (worst case) ranking result when candidates, including the correct one, shared equal MetFrag scores. Hence all potential isomers, e.g. double bond positional isomers, which usually have identical MetFrag scores, are covered and reported.

The original MetFrag scoring function considers the bond dissociation energy (BDE) of bonds which are cleaved during the *in silico fragmentation*. As the cleavage of C-C bonds of the fatty acid chains is unlikely to occur under the given conditions in the mass spectrometer, the BDE of this bond type was set to the arbitrarily high value of 10e9, which effectively eliminates fragments generated by a C-C cleavage.

### Lipid class specific classifiers for reliability calculation

A new feature of LipidFrag is the use of classifiers for reliability calculation of the MetFrag result. The distribution of the MetFrag raw scores depends on both the query spectra and the compound classes of the candidates, as shown in S1 Fig, S2 Fig and S3 Fig. Generally, in metabolomics this structural compound class classification is neither always obvious nor easy to obtain for small molecules, but for lipids there is the structural categorization initiated by the International Lipid Classification and Nomenclature Committee (ILCNC), available on the Lipid Maps website [4]. With this nomenclature, the structures are hierarchically ordered and encoded as positions in the Lipid Maps ID. This classification was used here to obtain well-defined ranges of MetFrag raw scores for particular lipid classes. Therefore, a training step was implemented to predict the reliability of MetFrag results based on the training of classifiers with MS/MS spectra of the lipids standard material for different lipid sub classes (S2–S6 Tables). For this task one classifier was created for each lipid subclass, where raw scores of correctly identified structures from the lipid standard materials served as foreground data. The same spectra were queried with deliberately wrong precursor candidates in the same mass range (up to 150 ppm), originating from the other lipid sub classes respectively, to obtain a decoy database and subsequently the MetFrag scores for the background data set. This approach was inspired from proteomics, where foreground and background training data are used to assign significance values to peptide identifications [25].

Gamma distributions were used to model the scores for the foreground and background data. The model parameters for the distributions were calculated by maximum-likelihood estimation on the fore- and background dataset. For each lipid class a separate classifier was trained, because the MetFrag scores exhibit large differences between the classes.

Eq (1) shows the calculation of the foreground class probabilities (FCP) of a MetFrag result with the bayesian approach, where P (score | Foreground, Θ) is the likelihood of the foreground model represented by a gamma distribution of the lipid sub class for the present score and *P (score | Background*, *Θ)* is the corresponding likelihood of the present score in the background model which is also represented by a gamma distribution. The estimated parameters of the distributions are represented by *Θ*.

For testing, a 10-fold cross-validation was applied. FCPs of the lipid classes were used to calculate the true positive and false negative rates on the test dataset to determine a Receiver Operating Characteristic curve (ROC) and the Area under Curve (AUC) as quality measure of the different classifiers.

$$FCP=\frac{p\left(score|Foreground,\theta \right)}{p\left(score|Foreground,\theta \right)+p\left(score|Background,\theta \right)}$$ (1)

### Reliability of MetFrag results

After training, the classifiers were used to predict the reliability of MetFrag candidate identifications for the *C*. *elegans* MS/MS spectra, where the correct candidate is unknown. Given a candidate list processed by MetFrag as SDF or CSV file, LipidFrag calculates the FCP for each candidate lipid in this result list by first selecting the appropriate classifier based on the candidate’s Lipid Maps ID. The selected classifier together with the calculated MetFrag raw score is used to calculate the FCP value. Those results, where no candidate exceeds a defined FCP threshold (of e.g. 0.95) have to be treated as unreliable or not identified.

### LipidBlast identification

For comparison lipid annotations were performed using the LipidBlast in silico tandem MS library [18]. The provided LipidBlast fork (v2 Hiroshi Tsugawa fork) was downloaded and converted by Lib2NIST tool (v1.0.4.38 (beta), options: “Include Synonyms”: Yes, “MW from chem. formula”: Yes, “MS/MS spectra only”: Yes, “2008 MS Search compatible”: Yes) to NIST format and used as spectral library for LipidBlast annotation of all standards used for LipidFrag available in MGF format obtained from Genedata Expressionist for MS 8.2. The NIST MSPepSearchGUI (v0.91, options: defaults except for “Q-TOF”: Yes, “Min. match factor”: 100, “Presearch mode”: Standard, “Load libraries in memory”: No, “Max. number of output hits”: 10, “Presearch mode”: Standard, “Precursor ion tolerance”: 0.02, “Fragment peak m/z tolerance”: 0.02) was used to process input spectra in batch mode. LipidMaps identifiers provided for the correct identifications were mapped to common names annotated by LipidBlast for comparison with the LipidFrag annotations. The pessimistic rankings (among the top 10 reported candidates) were calculated based on the Rev-Dot (reverse dot) scores and compared with the LipidFrag results.

### Availability of LipidFrag

LipidFrag comes with several R scripts available at https://github.com/c-ruttkies/LipidFrag together with the used data for training and an example for lipid class prediction. After prediction and model parameter training LipidFrag uses a MetFrag result CSV file retrieved by using MetFrag and the Lipid Maps database for candidate retrieval and predicts the underlying lipid class. The calculated FCP value is an indication of the reliability of the lipid identification. The newest version of the MetFrag commandline tool is available at http://c-ruttkies.github.io/MetFrag.

## Results

LipidFrag uses the result scores of a lipid candidate list retrieved from MetFrag, which performs *in silico* fragmentation of lipids. Then the matching classifier is selected based on the lipid sub class of a currently considered lipid candidate in the candidate list. Using the bayesian equation, LipidFrag calculates the posterior probability of the MetFrag score under the assumption to come from the foreground distribution of the selected bayesian classifier. This probability value can then be used as prediction of the lipid class of the regarded MS/MS spectrum, and secondly, as a measure of reliability of the current MetFrag lipid annotation to filter out false positive lipid assignments.

### Analysis of lipid standard materials

For the positive ion mode spectra, classifiers were built for the following lipid sub classes: PC (LMGP0101), PE (LMGP0201), PS (LMGP0301), PI (LMGP0601), Cer (LMSP0201/ LMSP0202) and TG (LMGL0301). As the scores for the Cer species (LMSP02) show a bimodal distribution in positive ion mode, two separate classifiers were trained for the available ceramide sub classes (LMSP0201 and LMSP0202) for the foreground data. Compared to a single classifier for the whole Ceramide main class, this captures the multimodal score ranges of the lipid sub classes in a better way (S1 Fig). For the negative ion mode spectra, the lipid sub classes: PC (LMGP0101), PE (LMGP0201), PS (LMGP0301), PG (LMGP0401), PI (LMGP0601) and Cer (LMSP0201/ LMSP0202) were used for training. As candidates for the LMGP0101 sub class show similar MetFrag scores on LMGP0201 sub class MS7MS spectra a combined classifier was trained. This resulted in six different classifiers for positive and five for negative ion mode. With these classifiers, used for positive and negative ion mode, LipidFrag is able to cover already over one third of the lipid species in the Lipid Maps database.

The classifiers were extensively cross-validated on the lipid standards spectra to generate receiver operating characteristic (ROC) curves and the corresponding area under curve (AUC) values as measure of identification performance. These values are partly shown in Table 1, for the full results see S1 Fig. For clarity, results are grouped into three lipid types: ceramides, glycerophospholipids and glycerolipids, and presented separately in the following paragraphs. Mean ranks shown in Table 1 are calculated with and without a FCP threshold to highlight the performance using the LipidFrag classifiers. To reduce the false negative rate a FCP threshold of 0.6 was set within LipidFrag. With this value the number of false positive assignments could be reduced from 91% to 57% for positive ion mode and from 93% to 27% for negative mode.

**Table 1:** Results of the MetFrag identification and the classifier testing.

Negative ion mode							
Metric	LMGL0301 (TG)	LMGP0101,LMGP0201 (PC, PE)	LMGP0201(PE)	LMGP0301 (PS)	LMGP0401 (PG)	LMGP0601(PI)	LMSP0201,LMSP0202 (Cer)
FCP+	—	0.871	—	0.979	0.888	0.834	0.817
FCP-	—	0.098	—	0.009	0.164	0.154	0.236
AUC	—	0.979	—	1.0	0.901	0.961	0.931
Mean Rank	—	2.2	—	1.8	1.8	2.6	1.3
Mean Rank (FCP > = 0.6)	—	2.3 (68%)	—	1.8 (55%)	1.8 (94%)	2.4 (78%)	1.2 (63%)
Cand	—	31.3	—	15.8	15.3	14.6	2.3
positive ion mode							
Metric	LMGL0301 (TG)	LMGP0101 (PC)	LMGP0201 (PE)	LMGP0301 (PS)	LMGP0401 (PG)	LMGP0601 (PI)	LMSP0201, LMSP0202 (Cer)
FCP+	1.000	0.551	0.994	0.969	—	1.000	0.908
FCP-	0.000	0.442	0.000	0.000	—	0.000	0.095
AUC	1.0	0.799	1.0	1.0	—	1.0	0.935
Mean Rank	3.1	5.8	1.7	1.9	—	1.0	1.17
Mean Rank (FCP > = 0.6) (FP-Rate)	1.7 (16%)	3.0 (45%)	1.7 (49%)	1.9 (9%)	—	1.0 (100%)	1.0 (68%)
Cand	33.9	26.5	26.5	14.9	—	15.3	2.2

The mean values of the FCPs retrieved from the cross-validation for the foreground (FCP+, higher is better) and the background (FCP-, lower is better) scores are shown. An AUC of 1.0 represents the best possible classification result for the corresponding lipid main/sub class. Additionally, the mean rank of the correct candidate (Rank) using MetFrag and LipidFrag with a FCP threshold of 0.6 together with the discarded proportion of false positives (FP-Rate) and the mean number of candidates retrieved (Cand) are given.

### Ceramides

Ceramides have quite distinct molecular formulas compared to other lipid classes (i.e. glycerophospholipids); therefore, the overlap with other classes and the number of potential candidates is low. Major differences between different ceramide species are the length of the sphingoid base, the number of hydroxyl groups in the sphingoid base, the length of the N-linked fatty acid and total number of double bonds. The fragmentation of ceramides has been studied extensively by Hsu et al. [26], focusing mainly on the [M-H]^-^ ions, whereas here ceramides were observed predominatly as [M+HCOO]^-^ adducts in negative ion mode. Both positive and negative ion modes were used to characterize the ceramides. In total, 17 ceramides were identified manually from obtained MS/MS, with 11 found in both ion modes, 2 in negative and 4 in positive ion mode only.

LipidFrag shows the best results for ceramides in positive ion mode, indicated by the AUCs of 0.935 for the sphingenine and sphinagine lipids (LMSP0201 /LMSP0202). In negative ion mode the AUC is also good with a value of 0.931. The mean rank of the correct solution is 1.17 in positive and 1.3 in negative ion mode, which is also due to the low number of candidates (see Table 1).

### Glycerophospholipids

Different classes of glycerophospholipids were subjected to fragmentation, including PC (LMGP0101), PE (LMGP0201), PS (LMGP0301), PG (LMGP0401) and PI (LMGP0601). The molecular formulas of PCs and PEs overlap considerably, which can lead to ambiguous results if only the accurate mass of the precursor is used for the annotation with potential structures. Ekroos et al. studied the use of fragmentation and fatty acid scanning using an ion trap MS for elucidation of the fatty acid composition of PCs [14]. Fragmentation is very class and ion mode specific, e.g. PCs yield mainly m/z 184.07 as the head group fragment in positive ion mode, whereas in negative ion mode fragments originating from [M+HCOO]^-^ adducts provide information about fatty acid composition and their positions. Diagnostic fragments indicating fatty acid composition were only detected for very high abundant species in positive ion mode. Several studies have shown that the carboxylate anion from the sn2 fatty acid is up to three times higher compared to sn1 [27]. PEs in contrast show mainly the diacylglycerol fragment derived from the neutral loss of the head group in positive mode and side chain fragments of very low intensity (usually below 2%). Therefore, MS3 of the diacylglycerol fragment is needed for side chain identification in positive ion mode. In negative ion mode, fragmentation of PE species yields carboxylate anions from sn1 and sn2 fatty acids similar to PCs.

Most of the glycerophospholipids show very good identification results with LipidFrag. This is indicated with the mean rank values 2.24, 1.8, 1.8 and 2.6 for the available PC/PE (LMGP0101 /LMGP0201), PS (LMGP0301), PG (LMGP0401) and PI (LMGP0601) species in negative ion mode. The AUCs of 0.979, 1.0, 0.901 and 0.961 also show excellent classification results (Table 1 and Fig 2).

In positive ion mode the PE (LMGP0201), PS (LMGP0301) and PI (LMGP0601) species show similar results with mean ranks of 1.7, 1.9 and 1.0 and the AUCs of 1.0. Though, the PC (LMGP0101) species show a similar performance with a mean rank of 1.69 when using a FCP filter with threshold 0.6 (see S8 Table) the filter sorted out 58 of the 71 spectra caused by the limited fragmentation which also indicated by a lower AUC of 0.799 (Table 1).

### Glycerolipids

Glycerolipids (LMGL0301) were detected in positive ion mode mainly as [M+NH4]^+^ adducts. From this adduct, typical fragmentation is the neutral loss of fatty acid side chains plus ammonia yielding a diacylglycerol-like fragment [28]. This loss can occur for all side chains and lead to a pattern that allows the identification of composition, but rarely provides sufficient information to determine the position of fatty acids in the intact lipid.

**Fig 3:**
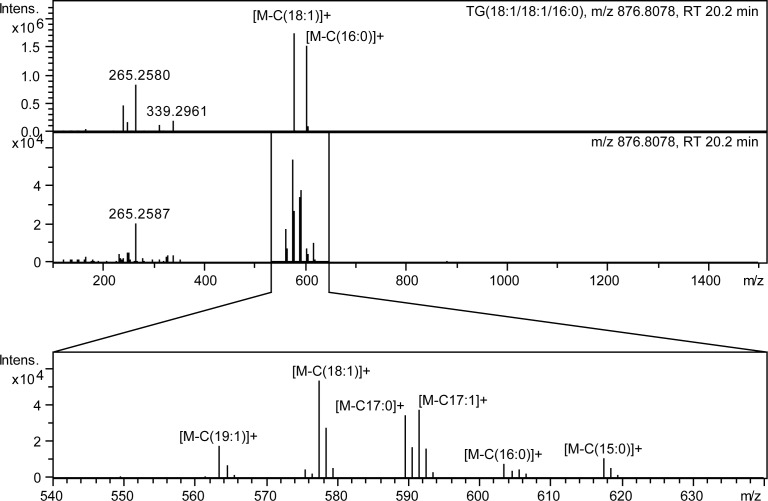
Example of co-elution and overlapping of different TG species in *C*. *elegans*. Analysis of spectra derived from TGs is complicated in real samples due to overlap of several isomeric and isobaric species. The upper panel shows the MS/MS spectrum of TG(18:1/18:1/16:0) standard and the lower of the same chromatographic peak in a *C*. *elegans* lipid extract.

Five different TG standards were employed as training data, showing previously known fragmentation pathways. These five compounds had different fatty acid compositions and therefore different retention times. However, in *C*. *elegans* samples many possible isomers and isobars are co-eluting with many different fatty acid combinations that can be deduced from fragmentation data (Fig 3). The TG species are observable in positive ion mode and the relating classifier shows a good result with an AUC of 1.0. However, the mean rank indicates a lower performance for the identification results with 3.1, as the typical loss of a fatty acid side chain during fragmentation is not only explained by the correct candidate, but also by structurally very similar TG species. The fragment peaks of these types of losses seem to be very specific for the lipid main class, indicated by the high AUC, but this does not help to distinguish between different TG lipids sharing the same molecular formula.

### Handling of mixed spectra

One problem not only for LipidFrag are non-pure spectra arising from co-isolation of co-eluting isomeric/isobaric lipids during the MS measurement. In order to test how well LipidFrag can deal with this, we created such spectra *in silico* using measured spectra as template. Overlap especially occurs for glycerolipids in the later elution range of the chromatogram, but might also occur for other lipids. Although the used UPLC method can separate major isobars of the glycerophospholipids [23], overlap might also occur with major interference most likely coming from isomers/isobars within same lipid class. Interference from different lipid classes having the same molecular formula can be neglected because polarity, and hence retention time is very different (e.g. PE(18:0/20:2) has a logP of 13.12, whereas the isobaric PC(18:0/17:0) has a logP of 11.47).

We used one measured lipid MS/MS as target and added interfering MS/MS peaks at the intensity ratios of 10:1, 2:1 and 1:1 and evaluated the MetFrag raw score of the true candidate. Mixtures included binary, ternary and even quaternary mixes of isobaric lipids (S1 Table). Results indicate that mixtures with an equal amount of target and interference cause a drop in the score and rank of the true candidate (S4 Fig) as expected.

The target substances still rank in the upper quarter. Results from one particular example in *C*. *elegans* samples having two isomeric PC species in on MS/MS spectrum are discussed in a later section (see Analysis of *C*. *elegans* samples).

### Analysis of publicly available MSMS spectra

To test the performance of the LipidFrag approach on an independent second dataset we used 415 negative ion mode lipid MS/MS spectra retrieved from Bio-MassBank [29] where a Lipid Maps ID was available for the correct candidate. Although these spectra were measured on a different instrument with higher mass error than the data used for classifier training, they served as an additional validation of the workflow. Altogether, the spectra were annotated by the submitters to be from ten different sub classes (LMGL0301, LMGP0101, LMGP0102, LMGP0103, LMGP0105, LMGP0201, LMGP0202, LMGP0203, LMGP0601 and LMSP0301). Table 2 shows the ranking results obtained from LipidFrag. The mean ranks within the lipid sub classes were 4.4, 6.0, 2.9, 3.9, 2.3, 2.8, 1.0, 1.0, 2.0, 1.8, respectively. Only two classifiers were available for the spectra originating from PC/PE (LMGP0101 /LMGP0201) and PI (LMGP0601) species. For the 180 MS/MS spectra 157 have been identified with the correct lipid sub class based on the foreground class probability (FCP) which is a true positive rate of ~87% for the low resolution spectra where a classifier was available. The LMGP0601 classifier calculated a sub class FCP which reached this threshold for all cases (9) and the LMGP0101/LMGP0201 classifier for 148 out of the 171 cases.

**Table 2:** LipidFrag rankings on the 415 Bio-MassBank spectra.

Lipid sub class	Mean rank	Median rank	Mean candidates	Median candidates	Number MS/MS
LMGL0301 (TG)	4.4	2.0	15.0	15.0	7
LMGP0101 (PC)	6.0	3.5	22.0	23.0	118
LMGP0102 (PC)	2.9	3.0	9,2	8.0	36
LMGP0103 (PC)	3.9	2.5	15.7	14.0	18
LMGP0105 (PC)	2.3	2.0	4.2	4.0	30
LMGP0201 (PE)	2.8	2.0	17.2	19.0	53
LMGP0202 (PE)	1.0	1.0	7.0	7.0	12
LMGP0203 (PE)	1.0	1.0	12.5	14.5	24
LMGP0601 (PI)	2.0	2.0	11.3	11.0	9
LMSP0301 (SM)	1.8	1.0	18.9	14.0	108
All	3.3	2.0	16.6	16.0	415

For each lipid sub class the number of MS/MS spectra available and the retrieved mean and median rank as well as the mean and median number of candidates are given.

### Comparison with LipidBlast annotations

The results of LipidBlast compared with the mean ranks of LipidFrag are shown in Table 3. The values indicate that results are comparable between both software tools. Nevertheless, there are slight deviations for some lipid classes and LipidFrag usually annotates more spectra (FCP threshold 0.6) for both ion modes.

**Table 3:** Comparison of LipidFrag with LipidBlast results.

Negative ion mode										
Mean Rank	TG (LMGL0301)	PC/PE (LMGP0101/LMGP0201)	PC (LMGP0101)		PE (LMGP0201)	PS (LMGP0301)		PG (LMGP0401)	PI (LMGP0601)	Cer (LMSP02/LMSP0202)
LipidFrag	—	2.3 (112)	—		—	1.8 (35)		1.8 (41)	2.4 (62)	1.2 (155)
LipidBlast	—	**1.2 (116)**	—		—	**1.0 (34)**		**1.0 (40)**	**2.3 (70)**	**1.0 (158)**
positive ion mode										
Mean Rank	TG (LMGL03)	PC/PE (LMGP0101/LMGP0201)	PC (LMGP01)	PE (LMGP02)		PS (LMGP03)	PG (LMGP04)		PI (LMGP06)	Cer (LMSP0201/LMSP0202)
LipidFrag	3.1 (25)	—	1.7 (13)	**1.7 (88)**		**1.9 (50)**	—		**1.0 (82)**	**1.0 (156)**
LipidBlast	**1.0 (13)**	—	**1.0 (9)**	1.8 (75)		7.8 (43)	—		NA (0)	**1.0 (158)**

The table shows the mean ranks of the used lipid main/sub classes in the standard data set. The LipidFrag results are calculated by using a FCP threshold of 0.6 (as in Table 1). Besides the mean rankings also the number of annotated spectra are given.

In positive ion mode on average LipidFrag could annotate 69 and LipidBlast 49.7 spectra across all lipid classes. Considering the mean ranks, LipidBlast showed better results for TG (LMGL0301) (1.0 to 3.1) species. No results were annotated for PI spectra as the predictions are missing in the current spectral database mirror of LipidBlast. Developers of LipidBlast indicated that predictions are in progress for several missing lipid classes and will be added to the library in the near future. LipidFrag showed better mean ranks for PE (LMGP0201) (1.7 to 1.8) and PS (LMGP0301) (1.9 to 7.8) species. Equal mean ranks for both software tool could be assigned to the Ceramide classes (LMSP0201 and LMSP0202) with a value of 1.0. Both software tools filtered out a large proportion of the PC spectra (LipidFrag: 58 spectra, LipidBlast: 62 spectra) as this lipid class shows sparse fragmentation in positive ion mode resulting in less informative MS/MS spectra.

For negative ion mode LipidFrag and LipidBlast could annotate an almost equal number of MS/MS spectra with mean values of 81 and 83.6 across all lipid classes. LipidBlast performed slightly better the Ceramide (LMSP0201) (1.0 to 1.6) and the PI (LMGP0601) species, whereas LipidFrag showed better mean ranks for PC/PE (LMGP0101 /LMGP0201) (2 to 2.3) and PG (LMGP0401) (1.0 to 1.8) species.

### Analysis of *C*. *elegans* samples

To demonstrate the applicability to biological data, lipids extracted from *C*. *elegans* were used, representing a realistic challenge for LipidFrag. The composition of the worm lipidome has been extensively reviewed [30]. Several lipid classes are present in the worm and different fatty acid combinations, including odd-numbered side chains, are possible in glycerol- and glycerophospholipids. Shotgun lipidomics was applied for analysis of a novel class of lipids only present in dauer larvae [31].

### Coverage of lipids with MS/MS spectra

**Fig 4:**
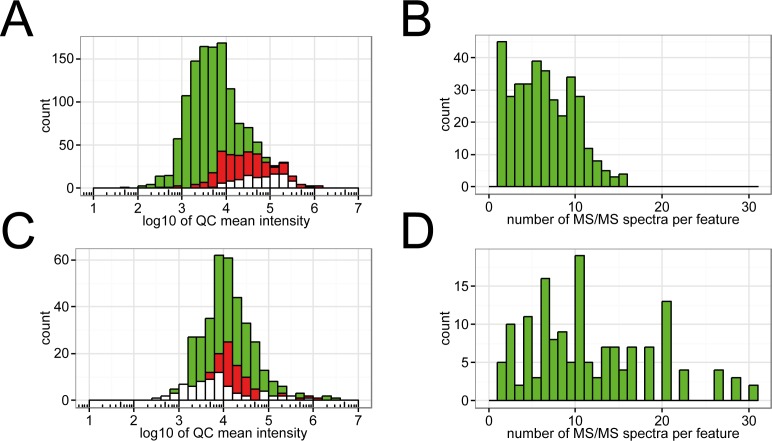
Histogram of intensities of features detected in positive ion mode. (A) The green histogram represents all features, in red are features with one or more associated MS/MS spectra and the white features having a FCP > 0.95 in LipidFrag. (B) Histogram of MS/MS spectra per feature in positive ion mode across all 5 technical replicates. (C) and (D) show the same for negative ion mode.

The total number of lipid features and those with at least one associated MS/MS spectrum are depicted in Fig 4. The green histogram shows all features, while the red one shows features with MS/MS spectra. Due to technical limitations of DDA, only a small fraction of the detected lipids is subjected to fragmentation, a problem well known from proteomics [32].

The DDA method used was able to fragment approximately a quarter (28%) of detected lipids. This number remains surprisingly constant, across different sample sets that have been analyzed with the same analytical method (data not shown). Different parameter settings for inclusion/exclusion lists and exclusion time have been tested. If the exclusion window is set too big, one or several features will be missed due to close elution of different isomeric and isobaric species, if too small, the same peak will be fragmented too often. Fig 4B and 4D show how often each peak with at least one MS/MS spectrum was fragmented across 5 technical replicates. Optimizing the analytical method for a better the coverage of peaks with MS/MS spectra is beyond the scope of this publication. However, 28% coverage corresponds to several thousand spectra (>3000), making the need for an automated analysis tool obvious.

### Application of LipidFrag workflow to *C*. *elegans* MS/MS spectra

LipidFrag then was applied to MS/MS spectra obtained from *C*. *elegans* lipid extracts. Table 3 gives an overview on detected lipid features in positive and negative ion mode runs. Altogether 1,518 MS/MS spectra acquired in negative and 2,355 MS/MS spectra in positive ion mode were processed. Results with a foreground class probability (FCP) of ≥ 0.95 can be found across the whole intensity range, although higher intensities seem to lead to better results in positive ion mode (Fig 4A). More important than precursor intensity is to detect diagnostic fragments, which especially is the case in negative ion mode, where fatty acyl side chains can be directly detected. Good results in this mode were also obtained for most of the middle intensity range (Fig 4C). Table 4 gives an overview on the number of detected lipid features and their corresponding MS/MS information and LipidFrag results.

**Table 4:** Overview on lipid MS1 features detected in *C*. *elegans* samples in the two respective ion modes with reliable LipidFrag results.

Ion mode	No. of cluster	With accurate mass annotation	With MS/MS	Manually identified in standards	Reliable LipidFrag (FCP cut-off)
Pos	1655	1297	685	65	• 108 (0.7) • 106 (0.8) • 100 (0.9) • 98 (0.95)
Neg	505	358	228	52	• 45 (0.7) • 43 (0.8) • 43 (0.9) • 40 (0.95)

For the 3,873 (1,518 + 2,355) *C*. *elegans* spectra used, the MetFrag *in silico* fragmentation and scoring took altogether ~31 hours (user+system time) on a single core CPU, i.e. 30 seconds per spectrum. Using the calculated classifiers, which are based on the standard lipid spectra, the FCP calculation for all 3,873 *C*. *elegans* spectra took less than 10 minutes, or 0.15 seconds per spectrum.

For the positive ion mode, LipidFrag detected 452 spectra as TG (LMGL0301), 69 as PE (LMGP0201). Additional 3 PE and 1 PC (LMGP0101) species were added by decreasing the FCP threshold to 0.9. In negative ion mode, LipidFrag found 206 spectra with PC/PE (LMGP0101/LMGP0201lipid sub class annotations having a FCP ≥ 0.95. With a lower FCP threshold of 0.9, additional 47 PC/ PE species were annotated. Irrespective of the ion mode over 22% of the LipidFrag results have a FCP ≥ 0.75 (S5 Fig).

**Fig 5:**
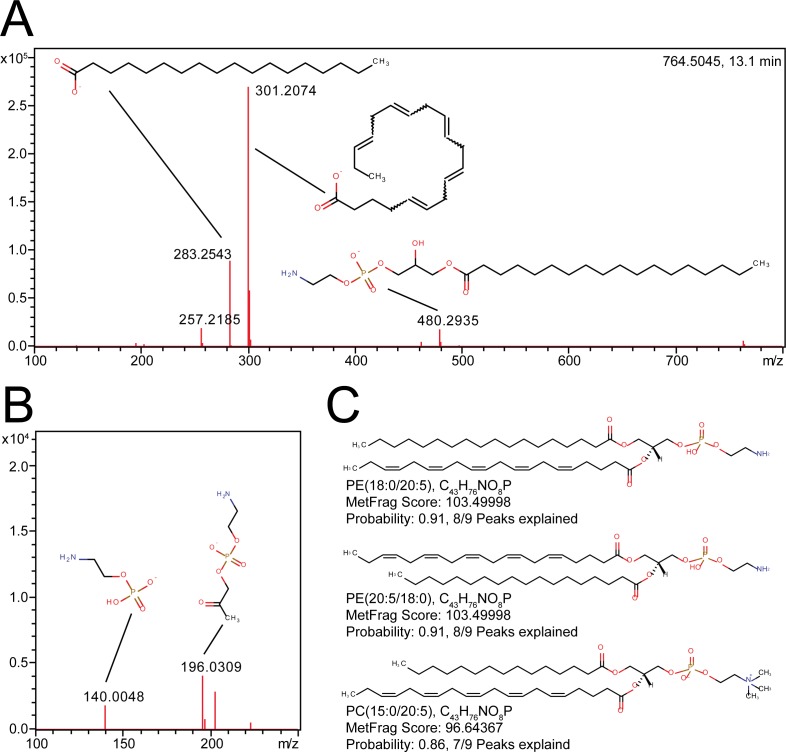
Example of a LipidFrag identification in *C*. *elegans* data. (A) MS/MS spectrum of m/z 764.5045 at 13.1 minutes detected in *C*. *elegans* with fragment structures annotated. (B) Close up of lower mass region (m/z 100–250). (C) Structures of the best three candidates obtained from MetFrag with result filtering using foreground class probabilities. Name, formula, MetFrag score and probability are indicated below each structure.

Fig 5 shows the spectrum of PE(18:0/20:5). The most prominent peaks show the corresponding fatty acids, with higher intensities for C20:5 bound at the sn2 position. A further diagnostic fragment [M-sn2-H]^-^ at m/z 480 is detected, and with lower intensities also the [M-sn1-H]^-^ at m/z 462 ion. Precursor mass together with these four peaks and their respective ratios allow manual identification as PE(18:0/20:5). Furthermore, the head group was detected as fragment together with a fragment containing the head group and the glycerol backbone. MetFrag was able to explain 8 of 9 fragments for identification. Additionally, small fragments derived from C20:5 were found. LipidFrag calculated a FCP of 0.91 for the result being a PE. The fatty acid positional isomer showed a similar score and probability. Because the scoring does not take any intensity ratios into account, both isomers obtain the same score. At this point, manual interpretation of intensities is required to determine which annotation is correct. The isobaric PC(15:0/20:5) was ranked third, with a similar MetFrag raw score (103.49998 for the correct PE and 96.64367 for the PC) but a lower FCP of 0.86 and only 7 of 9 peaks correctly explained. Number of explained peaks was used as additional metric for correct identification, in case scores and probabilities are similar. A second sample is depicted in S6 Fig and in the S6 and S7 Tables. At the respective retention time two PC isomers are coeluting, leading to a superposition of different MS/MS spectra. For the precursor ion two different possible annotations were found by MassTRIX, [M+HCOO]^-^ or [M-H]^-^. LipidFrag was able to correctly annotate both isomers using [M+HCOO]^-^ as precursor with high scores and FCPs (≥ 0.99), where 4 different isomers (two fatty acids and two positional isomers) were found on the first four ranks. Manual identification confirmed the automated results. In addition to the correct PC species, other PC and PE species were annotated due to several possibilities that arise from the merging of two lipids and other minor fatty acid fragments with very low intensities (e.g. C20:2) (S6 Table). On the other hand, results for the [M-H]^-^ annotation yielded only low scores and FCPs (< 0.1) (S7 Table). At the current stage no further details, e.g. on position of double bonds can be given without using specialized analytical approaches [33].

Both demonstrated that using MetFrag scores alone results can be ambiguous where the addition of the LipidFrag classifiers into the workflow improved automatic annotation results, by removing many false positive results (S8 and S9 Tables). Remaining spectra were either of low quality, low MetFrag scores or no training data was available due to missing standard materials for respective lipid class (e.g. glycosphingolipids, LMSP05). No classification could be made in the latter case.

Lipids in the used biological samples subjected to fragmentation by DDA were almost exclusively from the class of glycerophospholipids, di- and triacylglycerols. Lower concentration lipids, e.g. ceramides, were masked by these highly abundant classes. Using *C*. *elegans* lipid extracts it was shown that the developed approach can be applied to biological samples. Coverage of features with one or more associated MS/MS spectra has to be improved, e.g. using pseudo-targeted methods [34], data independent approaches and spectra reconstruction [35] or improved DDA [36]. Lastly, in order to achieve full lipidome coverage, several more classifiers for different lipid classes are needed, but not for all classes lipid standards (e.g. maradolipids detected in dauer larvae) are currently available.

## Discussion

Although the number of tools for automatic identification of lipids is increasing, most research still performs manual inspection of MS/MS spectra or automated comparison against rather small reference libraries. *In silico* fragmentation offers an elegant, automatic way to tentatively identify metabolites and lipids if no standard is available, by reducing the number of possible candidates or even propose just a single reliable match. A workflow was developed and validated for analysis of lipid MS/MS spectra derived from data dependent acquisition on a UPLC-Q-ToF-MS system. This workflow is based on annotation of potential lipids to the precursor mass, isotope clean-up of MS/MS spectra and identification using the *in silico* fragmentation tool MetFrag in combination with a novel reliability calculation based on bayesian classifiers. Lipid standard materials were used for validation purposes and the *in silico* analysis was compared against manual identification.

Cross-validation of the obtained results showed that the true correct identification can be easily separated from background spectra for most cases. Scores of correctly identified lipids and deliberately wrong candidates as decoys were used to generate fore- and background datasets to calculate the FCP giving a reliability of a result of an unknown to be correct. Using lipid standard materials, good performance of LipidFrag was shown, with high relative rankings of the correct candidate, high probabilities and high AUC values obtained from the cross-validation. Furthermore, comparison with LipidBlast, one of the most utilized tool for lipid spectra prediction, showed comparable results for both tools, with the main difference that the LipidFrag approach needs an initial training step for its classifiers but no *ab initio* information on fragmentation compared to LipidBlast. The workflow was applied to a lipid extract of *C*. *elegans*. From the obtained spectra, about 20% had high foreground class probabilities of ≥ 0.9. Higher identification rates could be achieved in future investigations by measuring more lipid standards from different classes to train more classifiers. However even with only 11 classifiers, the application of LipidFrag to MS/MS spectra derived from lipid extracts from *C*. *elegans* was successful and showed the advantage of this workflow.

An advantage here is that MetFrag does not rely on previously known fragmentation pathways and is therefore also applicable to novel lipid classes, currently not present in databases. In this case, candidate structures can be scored by generating potential structures, e.g. using theoretical lipids from LipidHome or even structures from a molecular structure generator like MOLGEN as input database [37].

For the results retrieved from the *C*. *elegans* data, comparison of the LipidFrag annotation with high probabilities and the manual identification for randomly-selected spectra showed excellent agreement with most of the peaks correctly explained by the *in silico* fragmentation. For application to complete lipidomics studies, the results from LipidFrag can serve as a first filtering and interpretation for further manual investigation, especially for potential marker peaks. A major limitation is co-elution of isomeric species leading to mixed MS/MS spectra. Although the chromatographic method is able to resolve several isomeric lipids as shown previously [23], not all of them can be resolved, especially for lipids like TGs where several isomers exist. Where identified spectra as training data are available, e.g. through authentic standards, LipidFrag can help in high-throughput identification. With the standard MS setups, as employed in this study, lipid class and fatty acid composition can be deduced. Our selected example with the PE(18:0/20:5) species from the biological dataset showed that the MetFrag score alone cannot distinguish ambiguous results. Here, the wrong candidate had a similar score to the correct one, but their FCPs were significantly different and enhanced the annotation confidence. Manual interpretation of obtained data often allows to additionally identify fatty acid position based on intensity ratios of fatty acid fragments, which is not possible with MetFrag.

Result output could be simplified by the lipid annotation scheme proposed by Liebisch et al. [38], which combines different lipid isomers under a common identifier. For mass spectrometry using UHR-Q-ToF-MS, the fatty acid scan level and fatty acid positional isomer are relevant. The former represents lipid identification of the fatty acid composition, but their position is not determined. This level is well suited for LipidFrag identification. For example, all isomeric results can be collapsed under a common identifier, which would be easier to interpret. Unfortunately, the Liebisch annotation is currently not widespread in structural databases. LipidHome is an *in silico* database [5], using this nomenclature, whereas no structural representation of the chemical structure is available, which would be needed for MetFrag. Currently, no chemoinformatics representation exists to encode ambiguity in the position of double bonds [13].

The use of data dependent fragmentation in conjunction with non-targeted studies can further benefit from improved chromatographic methods with increased chromatographic resolution, especially in regions where several lipids co-elute. Different column chemistry, e.g. C30 stationary phase, helps with isomer separation. In the end, a trade-off between resolution, analysis time and throughput has to be found. Here, only one particular extract was used to test the workflow, but in a more extensive study it is likely that more MS/MS spectra from different lipid features would be obtained, based on natural sample inhomogeneity and differences between sample groups. The new approach of all ion fragmentation or data independent acquisition (DIA) offered by most MS vendors can increase the coverage, but tools for deconvolution and reconstruction of MS/MS spectra from this type of acquisition are still very limited today [39, 40]. Additionally, positively identified lipids can be uploaded to general repositories, e.g. MassBank, to improve data distribution.

Here, this method is applied to different *C*. *elegans* studies and allows comprehensive analysis of the nematodes’ lipidome, but is also applicable datasets from different experiments.

## Conclusion

Our newly developed workflow LipidFrag improves lipid identification from simple annotation to higher levels of accuracy. It utilizes *in silico* fragmentation of lipid candidate structures. Fragments explained by LipidFrag match known fragmentation pathways, e.g. neutral losses of lipid headgroups and fatty acid side chain fragments. These *in silico* fragmentation results are used to determine reliability scores calculated by bayesian classifiers, which helps to distinguish between true and false annotation results. For training of the classifiers authentic chemical standards from known lipid classes were used. This novel, additional filter step decreases interference from isomeric or isobaric results from different lipid classes having similar MetFrag scores. Extensive cross-validation and application to lipids from *C*. *elegans* showed its applicability. With inclusion of more and more future available lipid standards identification rates using LipidFrag will increase.

## Supporting information

S1 Information A website http://msbi.ipb-halle.de/msbi/lipidfrag has been created to provide additional material for this manuscript. All files are provided for both positive and negative ion mode. The peaklist archives contain the actual MetFrag query files of the standard and *C*. *elegans* MS/MS spectra. Furthermore, the result files are attached containing the MetFrag identifications and LipidFrag’s calculated foreground class probabilities for the *C*. *elegans* peaklists.
(DOCX)

S1 Fig Histograms of MetFrag score distributions for positive ion mode. Histograms of back- (red) and foreground (green) datasets with their respective modeled distributions from specific lipid sub-classes.
(TIF)

S2 Fig Histograms of MetFrag score distributions for negative ion mode. Histograms show back- (red) and foreground (green) datasets with their modeled distributions from specific lipid sub-classes.
(TIF)

S3 Fig Scatterplots of raw MetFrag scores from lipid standard material MS/MS spectra. The score are shown for negative (A) and positive (B) ion mode.
(TIF)

S4 Fig MetFrag results from overlapping experiment. Rank as function of different mixtures is shown.
(TIF)

S5 Fig LipidFrag results on *C*. *elegans* data. The maximal foreground class probabilities (FCPs) and their histogramms calculated by LipidFrag are plotted in descending order for 2,355 MS/MS spectra in positive (A) and 1,518 MS/MS spectra in negative (B) ion mode originating from the C. elegans lipid extract.
(TIF)

S6 Fig LipidFrag annotation example from *C*. *elegans* dataset. (A) Extracted ion chromatogram of an example lipid and one MS/MS spectrum acquired at 13.11 minutes. Under this peak two isomeric PC species are co-eluting. LipidFrag identified all four isomer (fatty acid isomers and positional isomers) with high scores and probabilities (S6 Table). (B) MS/MS spectrum at 13.11 showing a mixed spectrum of two isomeric PC species.
(TIF)

S1 Table Target lipids and used interfering species for overlapping experiments. (PDF)

S2 Table Statistics on training MS/MS spectra from positive ion mode. (PDF)

S3 Table Statistics on training MS/MS spectra from negative ion mode. (PDF)

S4 Table Number of used MS/MS spectra for training in positive ion mode. (PDF)

S5 Table Number of used MS/MS spectra for training in negative ion mode. (PDF)

S6 Table LipidFrag results for *C*. *elegans* MS/MS spectrum shown in S6 Fig derived from [M+HCOO]^-^ annotation. (PDF)

S7 Table LipidFrag results for C. *elegans* MS/MS spectrum shown in S6 Fig derived from [M-H]^-^ annotation. (PDF)

S8 Table LipidFrag’s improvement of ranks for training MS/MS spectra in positive ion mode. (PDF)

S9 Table LipidFrag’s improvement of ranks for training MS/MS spectra in negative ion mode. (PDF)
