# Supplementary material for: LipidFrag: Improving reliability of in silico fragmentation of lipids and application to the Caenorhabditis elegans lipidome
Source: PLoS One. 2017 Mar 9;12(3):e0172311. doi: 10.1371/journal.pone.0172311 (PMC5344313; doi:10.1371/journal.pone.0172311)
Supplement: S1 Information — All files are provided for both positive and negative ion mode. The peaklist archives contain the actual MetFrag query files of the standard and C. elegans MS/MS spectra. Furthermore, the result files are attached containing the MetFrag identifications and LipidFrag’s calculated foreground class probabilities for the C. elegans peaklists. (DOCX) [file pone.0172311.s001.docx]

S1 Information. Supporting information is available online. A website http://msbi.ipb-halle.de/msbi/lipidfrag has been created to provide additional material for this manuscript. All files are provided for both positive and negative ion mode. The peaklist archives contain the actual MetFrag query files of the standard and *C. elegans* MS/MS spectra. Furthermore, the result files are attached containing the MetFrag identifications and LipidFrag’s calculated foreground class probabilities for the *C. elegans* peaklists.
