# Supplementary figures and images for: LipidFrag: Improving reliability of in silico fragmentation of lipids and application to the Caenorhabditis elegans lipidome
Source: PLoS One. 2017 Mar 9;12(3):e0172311. doi: 10.1371/journal.pone.0172311 (PMC5344313; doi:10.1371/journal.pone.0172311)

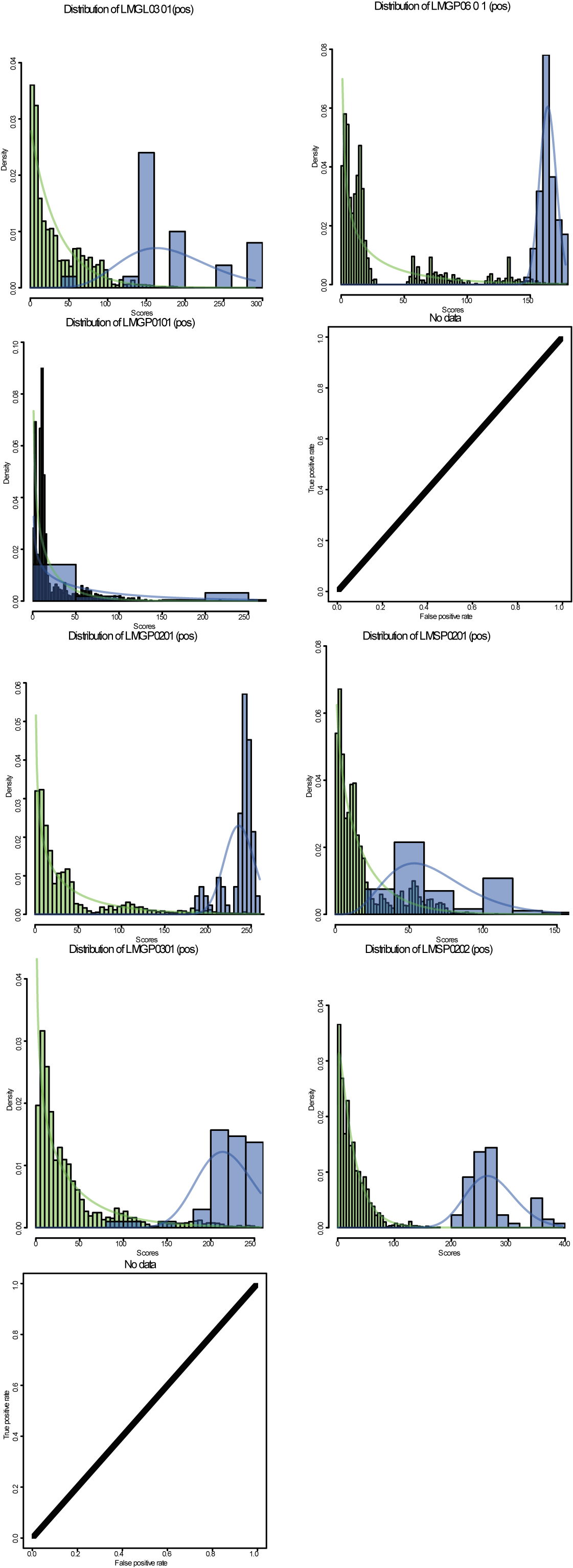

Supplement: S1 Fig — Histograms of back- (red) and foreground (green) datasets with their respective modeled distributions from specific lipid sub-classes. (TIF) [file pone.0172311.s002.tif]

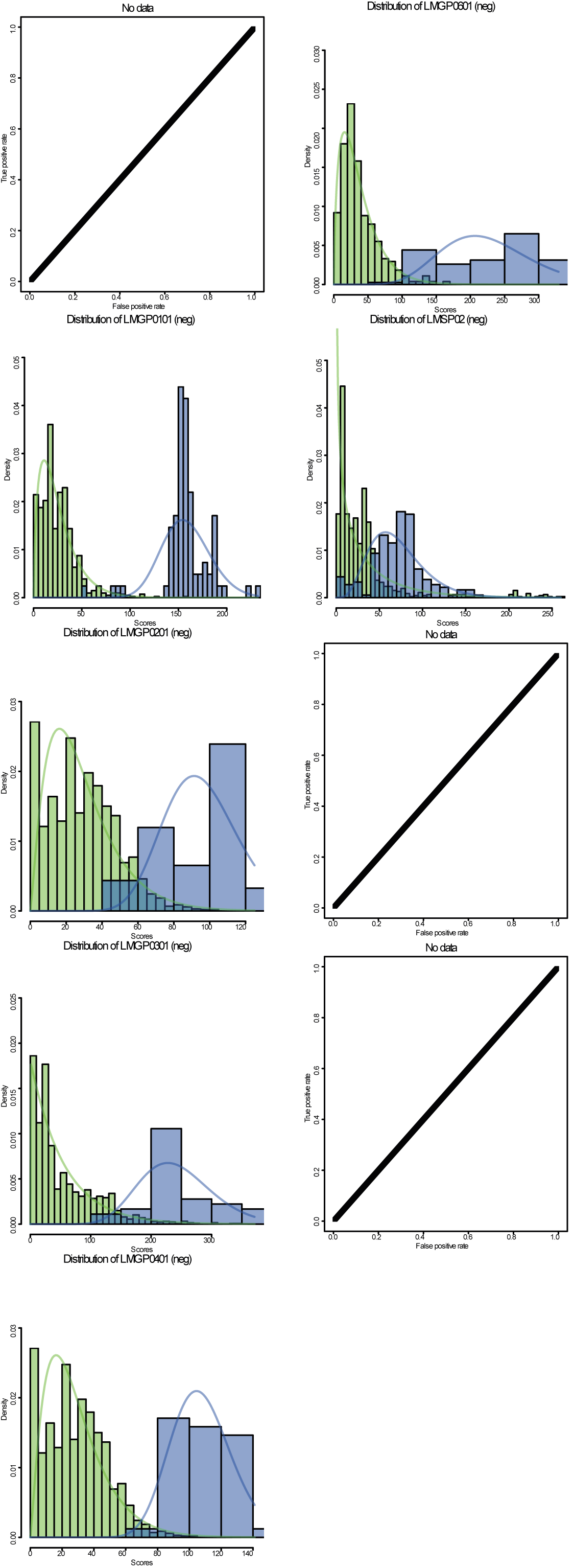

Supplement: S2 Fig — Histograms show back- (red) and foreground (green) datasets with their modeled distributions from specific lipid sub-classes. (TIF) [file pone.0172311.s003.tif]

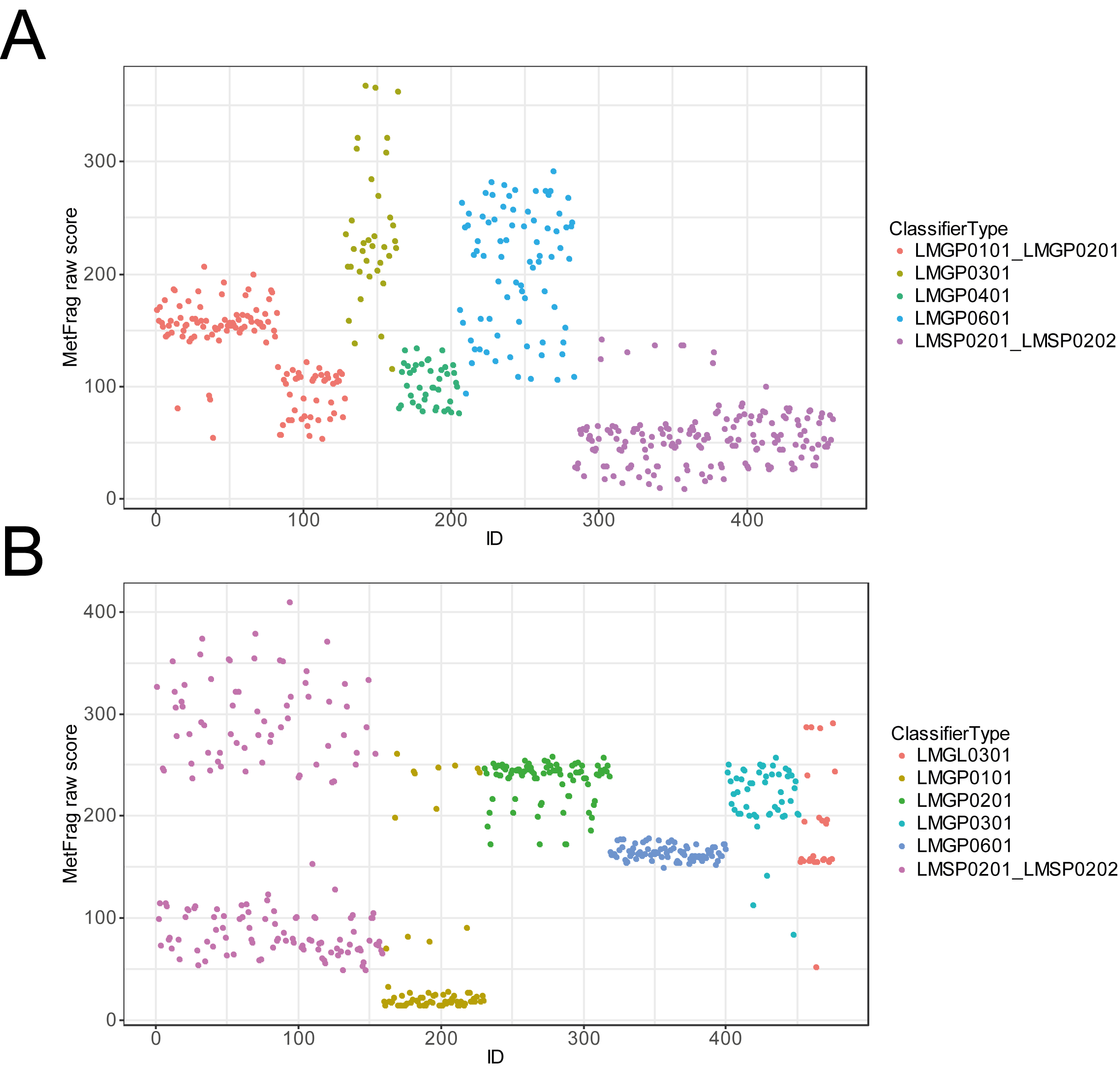

Supplement: S3 Fig — The score are shown for negative (A) and positive (B) ion mode. (TIF) [file pone.0172311.s004.tif]

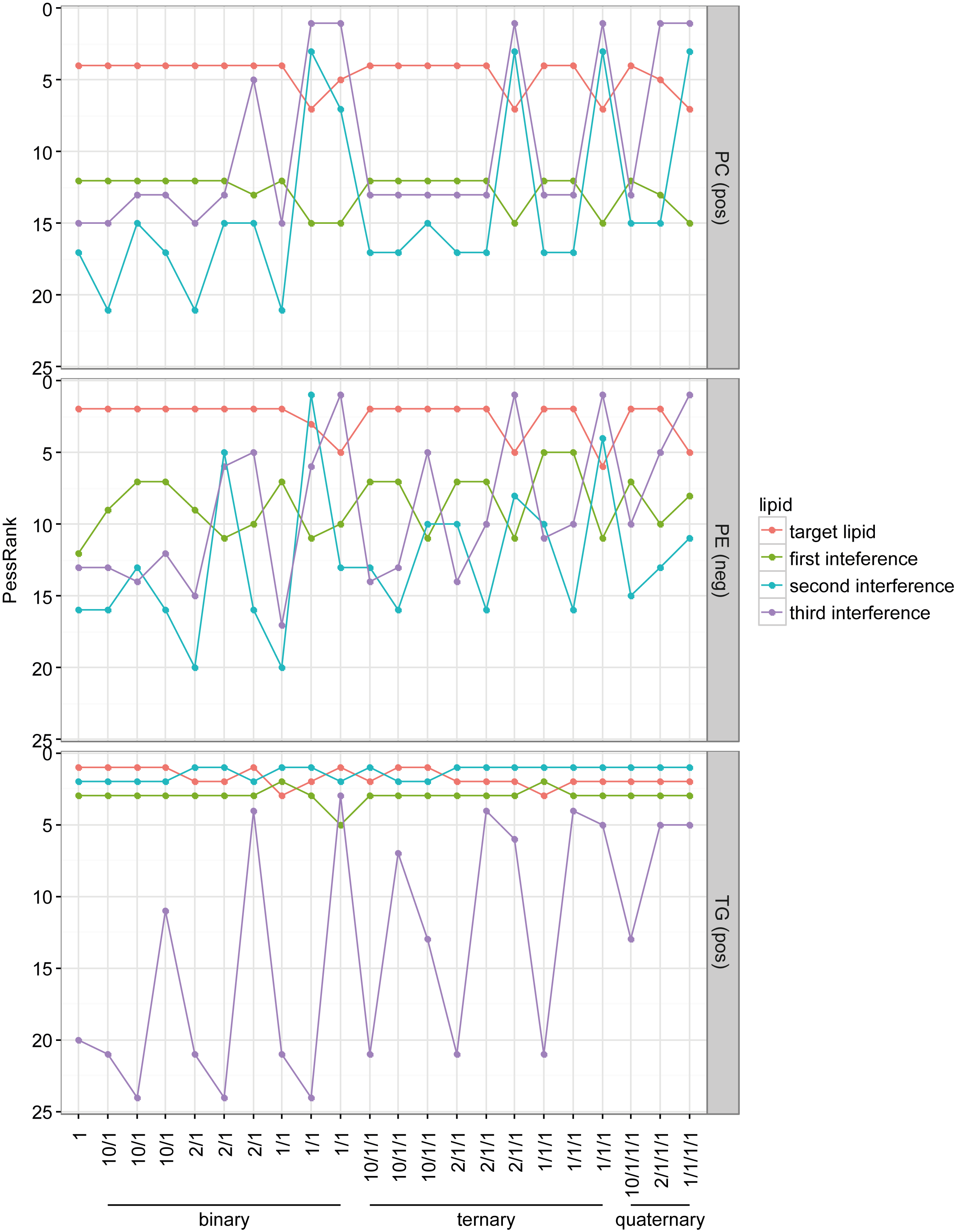

Supplement: S4 Fig — Rank as function of different mixtures is shown. (TIF) [file pone.0172311.s005.tif]

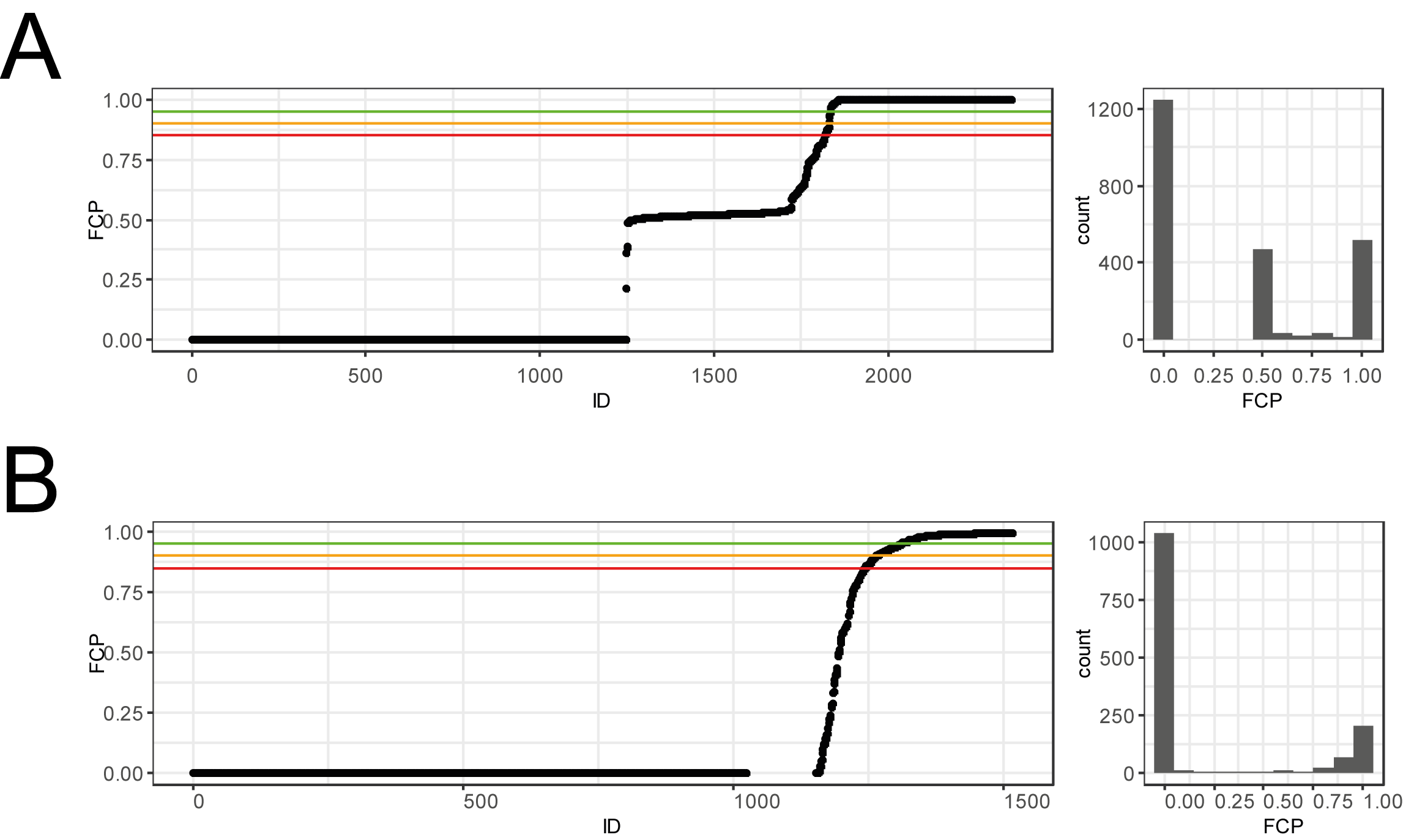

Supplement: S5 Fig — The maximal foreground class probabilities (FCPs) and their histogramms calculated by LipidFrag are plotted in descending order for 2,355 MS/MS spectra in positive (A) and 1,518 MS/MS spectra in negative (B) ion mode originating from the C. elegans lipid extract. (TIF) [file pone.0172311.s006.tif]

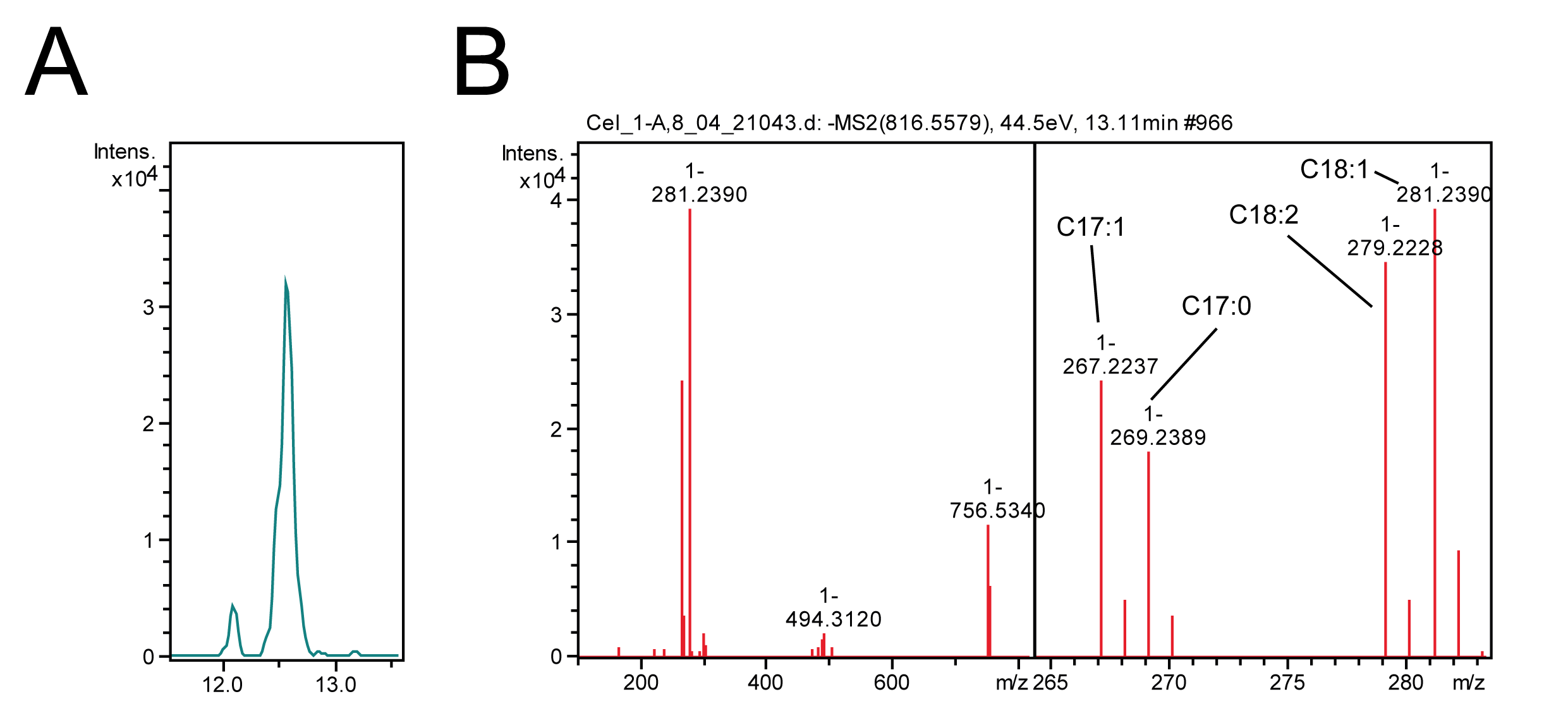

Supplement: S6 Fig — (A) Extracted ion chromatogram of an example lipid and one MS/MS spectrum acquired at 13.11 minutes. Under this peak two isomeric PC species are co-eluting. LipidFrag identified all four isomer (fatty acid isomers and positional isomers) with high scores and probabilities (S6 Table). (B) MS/MS spectrum at 13.11 showing a mixed spectrum of two isomeric PC species. (TIF) [file pone.0172311.s007.tif]
