# Supplementary material for: LipidFrag: Improving reliability of in silico fragmentation of lipids and application to the Caenorhabditis elegans lipidome
Source: PLoS One. 2017 Mar 9;12(3):e0172311. doi: 10.1371/journal.pone.0172311 (PMC5344313; doi:10.1371/journal.pone.0172311)
Supplement: S1 Table — (PDF) [file pone.0172311.s008.pdf]

S1 Table. *Target lipids and used interfering species for overlapping experiments.*

Target	*Interference*	*Ion mode*
PE(18:0/20:5)	*PE(18:1/20:4), PE(16:0/22:5), PE(16:1/22:4)*	*neg*
TG(18:1/18:1/16:0)	*TG(18:1/18:0/16:1), TG(18:2/18:0/16:0), TG(20:2/16:0/16:0)*	*pos*
PC(18:0/20:4)	*PC(18:1/20:3), PC(16:0/22:4), PC(16:1/22:2)*	*pos*
PE(18:0/18:2)	*PC(15:0/18:2)*	*pos*
