# Supplementary material for: LipidFrag: Improving reliability of in silico fragmentation of lipids and application to the Caenorhabditis elegans lipidome
Source: PLoS One. 2017 Mar 9;12(3):e0172311. doi: 10.1371/journal.pone.0172311 (PMC5344313; doi:10.1371/journal.pone.0172311)
Supplement: S2 Table — (PDF) [file pone.0172311.s009.pdf]

S2 Table. *Statistics on training MS/MS spectra from positive ion mode.*

	LMGP0101 (PC)	*LMGP0201 (PE)*	*LMGP0301 (PS)*	*LMGP0601 (PI)*	*LMGL0301 (TG)*	*LMSP0201 LMSP0202 (Cer)*
Number of MS2 spectra used for training	*71*	*88*	*51*	*82*	*25*	*172*
Mean number of informative MS2 peaks	*5.96*	*17.26*	*14.53*	*16.22*	*60.76*	*12.44*
Median number of informative MS2 peaks	*2*	*19*	*9*	*13*	*25*	*7*
Standard deviation of informative MS2 peaks	*7.22*	*8.34*	*12.47*	*11.94*	*51.85*	*13.15*
