# Supplementary material for: LipidFrag: Improving reliability of in silico fragmentation of lipids and application to the Caenorhabditis elegans lipidome
Source: PLoS One. 2017 Mar 9;12(3):e0172311. doi: 10.1371/journal.pone.0172311 (PMC5344313; doi:10.1371/journal.pone.0172311)
Supplement: S3 Table — (PDF) [file pone.0172311.s010.pdf]

S3 Table. *Statistics on training MS/MS spectra from negative ion mode.*

	LMGP0101 LMGP0201 (PC, PE)	*LMGP0301 (PS)*	*LMGP0401 (PG)*	*LMGP0601 (PI)*	*LMSP0201 LMSP0202 (Cer)*
Number of MS2 spectra used for training	*128*	*36*	*41*	*78*	*189*
Mean number of informative MS2 peaks	*7.01*	*6.81*	*8.98*	*11.73*	*9.40*
Median number of informative MS2 peaks	*7*	*7*	*9*	*13*	*10*
Standard deviation of informative MS2 peaks	*2.26*	*1.74*	*2.60*	*2.88*	*3.15*
