# Supplementary material for: LipidFrag: Improving reliability of in silico fragmentation of lipids and application to the Caenorhabditis elegans lipidome
Source: PLoS One. 2017 Mar 9;12(3):e0172311. doi: 10.1371/journal.pone.0172311 (PMC5344313; doi:10.1371/journal.pone.0172311)
Supplement: S4 Table — (PDF) [file pone.0172311.s011.pdf]

S4 Table. *Number of used MS/MS spectra for training in positive ion mode.*

Liebisch	*LMID*	*Number MS/MS spectra*
Cer(16:0_d18:0)	LMSP02020001	20
Cer(16:0_d18:1)	LMSP02010004	13
Cer(17:0_d18:1)	LMSP02010020	9
Cer(18:0_d18:1)	LMSP02010006	26
Cer(d18:0_18:0)	LMSP02020008	20
Cer(d18:0_22:0)	LMSP02020010	5
Cer(d18:0_24:0)	LMSP02020012	7
Cer(d18:0_24:1)	LMSP02020011	14
Cer(d18:1_22:0)	LMSP02010008	18
Cer(d18:1_24:0)	LMSP02010012	17
Cer(d18:1_24:1)	LMSP02010009	23
PC(12:0_20:2)	LMGP01011835	5
PC(14:0_20:3)	LMGP01012192	4
PC(16:0_16:0)	LMGP01010564	7
PC(16:0_16:1)	LMGP01010566	4
PC(16:0_20:5)	LMGP01010633	9
PC(16:0_22:6)	LMGP01012137	6
PC(17:0_18:1)	LMGP01010711	4
PC(18:0_18:1)	LMGP01010751	10
PC(18:0_22:5)	LMGP01010818	9
PC(18:0_22:6)	LMGP01010823	13
PE(12:0_18:0)	LMGP02011261	3
PE(12:0_18:1)	LMGP02011260	1
PE(14:0_16:0)	LMGP02010302	1
PE(14:1_16:0)	LMGP02010506	7
PE(16:0_16:1)	LMGP02011228	8
PE(16:0_17:1)	LMGP02011226	12
PE(16:0_18:1)	LMGP02010010	13
PE(16:0_19:1)	LMGP02010509	13
PE(17:1_18:1)	LMGP02010641	15
PE(18:1_18:1)	LMGP02010052	15
PI(16:0_18:1)	LMGP06010933	12
PI(16:0_18:2)	LMGP06010959	22
PI(16:0_18:3)	LMGP06010877	7
PI(18:0_18:1)	LMGP06010957	5
PI(18:0_18:2)	LMGP06010956	9
PI(18:0_18:3)	LMGP06010375	5
PI(18:1_18:2)	LMGP06010931	12
PI(18:2_18:2)	LMGP06010927	10
PS(18:0_18:1)	LMGP03010025	11
PS(18:0_20:4)	LMGP03010039	13
PS(18:0_22:4)	LMGP03010883	10
PS(18:0_22:6)	LMGP03010040	11
PS(18:1_18:1)	LMGP03010030	6
TG(14:0_14:0_14:0)	LMGL03012616	13
TG(16:0_18:2_18:2)	LMGL03010141	12
