# Supplementary material for: LipidFrag: Improving reliability of in silico fragmentation of lipids and application to the Caenorhabditis elegans lipidome
Source: PLoS One. 2017 Mar 9;12(3):e0172311. doi: 10.1371/journal.pone.0172311 (PMC5344313; doi:10.1371/journal.pone.0172311)
Supplement: S5 Table — (PDF) [file pone.0172311.s012.pdf]

S5 Table. *Number of used MS/MS spectra for training in negative ion mode.*

Liebisch	***LMID***	***Number MS/MS spectra***
Cer(16:0_d18:0)	LMSP02020001	15
Cer(16:0_d18:1)	LMSP02010004	21
Cer(d18:0_18:0)	LMSP02020008	17
Cer(d18:0_18:1)	LMSP02020015	30
Cer(d18:0_22:0)	LMSP02020010	9
Cer(d18:0_24:0)	LMSP02020012	10
Cer(d18:0_24:1)	LMSP02020011	14
Cer(d18:1_22:0)	LMSP02010008	26
Cer(d18:1_24:0)	LMSP02010012	19
Cer(d18:1_24:1)	LMSP02010009	28
PC(16:0_16:1)	LMGP01010566	11
PC(16:0_18:1)	LMGP01010581	10
PC(16:0_20:4)	LMGP01012136	13
PC(16:0_22:5)	LMGP01010647	11
PC(16:0_22:6)	LMGP01012137	8
PC(18:0_18:1)	LMGP01010761	10
PC(18:0_20:4)	LMGP01012144	11
PC(18:1_18:1)	LMGP01010855	4
PC(18:1_18:2)	LMGP01012149	4
PE(16:0_16:1)	LMGP02011228	7
PE(16:0_17:1)	LMGP02011226	11
PE(16:0_18:1)	LMGP02010311	13
PE(17:1_17:1)	LMGP02011209	2
PE(18:1_18:1)	LMGP02010109	7
PE(18:1_19:1)	LMGP02010646	6
PG(16:0_16:1)	LMGP04010910	10
PG(16:0_17:1)	LMGP04010908	10
PG(16:0_18:1)	LMGP04010987	10
PG(16:0_19:1)	LMGP04010196	1
PG(17:1_18:1)	LMGP04010328	6
PG(18:1_18:1)	LMGP04010985	4
PI(16:0_18:1)	LMGP06010001	11
PI(16:0_18:2)	LMGP06010959	13
PI(16:0_18:3)	LMGP06010944	11
PI(18:0_18:2)	LMGP06010956	14
PI(18:0_18:3)	LMGP06010284	19
PI(18:2_18:2)	LMGP06010927	10
PS(18:0_18:1)	LMGP03010025	7
PS(18:0_20:4)	LMGP03010039	8
PS(18:0_22:4)	LMGP03010883	12
PS(18:1_18:1)	LMGP03010030	9
