# Supplementary material for: LipidFrag: Improving reliability of in silico fragmentation of lipids and application to the Caenorhabditis elegans lipidome
Source: PLoS One. 2017 Mar 9;12(3):e0172311. doi: 10.1371/journal.pone.0172311 (PMC5344313; doi:10.1371/journal.pone.0172311)
Supplement: S6 Table — (PDF) [file pone.0172311.s013.pdf]

**S6 Table.** *LipidFrag results for C. elegans MS/MS spectrum shown in S6 Fig derived from [M+HCOO]⁻ annotation.*

| *Identifier* | *FragmenterScore* | *ForeProb* | *BackProb* | *FCP* | *LipidMapsClass* | *Liebisch* | *CommonName* |
|---|---|---|---|---|---|---|---|
| LMGP01011601 | 211.078536 | 0.00071437 | 4.22E-06 | 0.9941219 | LMGP0101_LMGP0201 | PC(17:1_18:1) | PC(18:1(9Z)/17:1(9Z)) |
| LMGP01011532 | 211.078536 | 0.00071437 | 4.22E-06 | 0.9941219 | LMGP0101_LMGP0201 | PC(17:1_18:1) | PC(17:1(9Z)/18:1(9Z)) |
| LMGP01011621 | 204.858186 | 0.00090666 | 5.59E-06 | 0.99386996 | LMGP0101_LMGP0201 | PC(17:0_18:2) | PC(18:2(9Z,12Z)/17:0) |
| LMGP01011505 | 204.858186 | 0.00090666 | 5.59E-06 | 0.99386996 | LMGP0101_LMGP0201 | PC(17:0_18:2) | PC(17:0/18:2(9Z,12Z)) |
| LMGP01011839 | 187.446603 | 0.00166632 | 1.22E-05 | 0.99271481 | LMGP0101_LMGP0201 | PC(15:0_20:2) | PC(20:2(11Z,14Z)/15:0) |
| LMGP01011422 | 187.446603 | 0.00166632 | 1.22E-05 | 0.99271481 | LMGP0101_LMGP0201 | PC(15:0_20:2) | PC(15:0/20:2(11Z,14Z)) |
| LMGP01011561 | 178.884598 | 0.00216956 | 1.79E-05 | 0.9918015 | LMGP0101_LMGP0201 | PC(17:2_18:0) | PC(17:2(9Z,12Z)/18:0) |
| LMGP01011587 | 178.884597 | 0.00216956 | 1.79E-05 | 0.9918015 | LMGP0101_LMGP0201 | PC(17:2_18:0) | PC(18:0/17:2(9Z,12Z)) |
| LMGP02010329 | 176.274435 | 0.00233929 | 2.01E-05 | 0.99146 | LMGP0101_LMGP0201 | PE-NMe2(18:1_18:1) | PE-NMe2(18:1(9E)/18:1(9E)) |
| LMGP02010326 | 176.274435 | 0.00233929 | 2.01E-05 | 0.99146 | LMGP0101_LMGP0201 | PE-NMe2(18:1_18:1) | PE-NMe2(18:1(9Z)/18:1(9Z)) |
| LMGP01011487 | 175.537514 | 0.00238849 | 2.08E-05 | 0.99135738 | LMGP0101_LMGP0201 | PC(16:1_19:1) | PC(16:1(9Z)/19:1(9Z)) |
| LMGP01011763 | 175.537514 | 0.00238849 | 2.08E-05 | 0.99135738 | LMGP0101_LMGP0201 | PC(16:1_19:1) | PC(19:1(9Z)/16:1(9Z)) |
| LMGP01011810 | 175.114933 | 0.00241695 | 2.12E-05 | 0.99129723 | LMGP0101_LMGP0201 | PC(15:1_20:1) | PC(20:1(11Z)/15:1(9Z)) |
| LMGP01011451 | 175.114933 | 0.00241695 | 2.12E-05 | 0.99129723 | LMGP0101_LMGP0201 | PC(15:1_20:1) | PC(15:1(9Z)/20:1(11Z)) |
| LMGP01012036 | 158.299794 | 0.00365374 | 4.48E-05 | 0.98789463 | LMGP0101_LMGP0201 | PC(13:0_22:2) | PC(22:2(13Z,16Z)/13:0) |
| LMGP01011360 | 158.299794 | 0.00365374 | 4.48E-05 | 0.98789463 | LMGP0101_LMGP0201 | PC(13:0_22:2) | PC(13:0/22:2(13Z,16Z)) |
| LMGP02010669 | 41.6127964 | 4.30E-05 | 0.00548433 | 0.00777344 | LMGP0101_LMGP0201 | PE(18:2_20:0) | PE(18:2(9Z,12Z)/20:0) |

| | | | | | | | |
|---|---|---|---|---|---|---|---|
| LMGP02010125 | 41.6127964 | 4.30E-05 | 0.00548433 | 0.00777344 | LMGP0101_LMGP0201 | PE(18:2_20:0) | PE(20:0/18:2(9Z,12Z)) |
| LMGP02010882 | 21.6291516 | 1.50E-07 | 0.00972995 | 1.54E-05 | LMGP0101_LMGP0201 | PE(18:0_20:2) | PE(20:2(11Z,14Z)/18:0) |
| LMGP02010124 | 21.6291516 | 1.50E-07 | 0.00972995 | 1.54E-05 | LMGP0101_LMGP0201 | PE(18:0_20:2) | PE(18:0/20:2(11Z,14Z)) |
| LMGP02010811 | 19.9674938 | 7.00E-08 | 0.01005307 | 6.96E-06 | LMGP0101_LMGP0201 | PE(19:1_19:1) | PE(19:1(9Z)/19:1(9Z)) |
| LMGP02010853 | 15.9409135 | 7.65E-09 | 0.01068161 | 7.16E-07 | LMGP0101_LMGP0201 | PE(18:1_20:1) | PE(20:1(11Z)/18:1(9Z)) |
| LMGP02010126 | 15.9409135 | 7.65E-09 | 0.01068161 | 7.16E-07 | LMGP0101_LMGP0201 | PE(18:1_20:1) | PE(18:1(9Z)/20:1(11Z)) |
| LMGP02010615 | 9.21007894 | 2.61E-11 | 0.01068391 | 2.44E-09 | LMGP0101_LMGP0201 | PE(17:2_21:0) | PE(17:2(9Z,12Z)/21:0) |
| LMGP02010998 | 9.2100785 | 2.61E-11 | 0.01068391 | 2.44E-09 | LMGP0101_LMGP0201 | PE(17:2_21:0) | PE(21:0/17:2(9Z,12Z)) |
| LMGP02010536 | 7.54916403 | 3.09E-12 | 0.01029313 | 3.01E-10 | LMGP0101_LMGP0201 | PE(16:1_22:1) | PE(16:1(9Z)/22:1(11Z)) |
| LMGP02011047 | 7.54916387 | 3.09E-12 | 0.01029313 | 3.01E-10 | LMGP0101_LMGP0201 | PE(16:1_22:1) | PE(22:1(11Z)/16:1(9Z)) |
| LMGP02010122 | 6.96075023 | 1.29E-12 | 0.01009448 | 1.27E-10 | LMGP0101_LMGP0201 | PE(18:0_20:2) | PE(20:2(5Z,8Z)/18:0) |
| LMGP02011077 | 0.68295293 | 5.84E-24 | 0.00348485 | 1.67E-21 | LMGP0101_LMGP0201 | PE(16:0_22:2) | PE(22:2(13Z,16Z)/16:0) |
| LMGP02010513 | 0.68295293 | 5.84E-24 | 0.00348485 | 1.67E-21 | LMGP0101_LMGP0201 | PE(16:0_22:2) | PE(16:0/22:2(13Z,16Z)) |
