# Supplementary material for: LipidFrag: Improving reliability of in silico fragmentation of lipids and application to the Caenorhabditis elegans lipidome
Source: PLoS One. 2017 Mar 9;12(3):e0172311. doi: 10.1371/journal.pone.0172311 (PMC5344313; doi:10.1371/journal.pone.0172311)
Supplement: S7 Table — (PDF) [file pone.0172311.s014.pdf]

S7 Table. *LipidFrag results for C. elegans MS/MS spectrum shown in S6 Fig derived from $[M-H]^-$ annotation.*

Identifier	*FragmenterScore*	*ForeProb*	*BackProb*	*FCP*	*LipidMapsClass*	*Liebisch*	*CommonName*
LMGP03010683	31.8777846	1.06E-09	0.00964949	1.09E-07	LMGP0301	PS(17:1_21:0)	PS(21:0/17:1(9Z))
LMGP03010272	31.8777846	1.06E-09	0.00964949	1.09E-07	LMGP0301	PS(17:1_21:0)	PS(17:1(9Z)/21:0)
LMGP03010961	13.6331169	2.52E-14	0.00560772	4.49E-12	LMGP0301	PS(18:0_20:1)	PS(18:0/20:1(11Z))
LMGP03010539	13.6331169	2.52E-14	0.00560772	4.49E-12	LMGP0301	PS(18:0_20:1)	PS(20:1(11Z)/18:0)
LMGP03010957	11.9934803	4.71E-15	0.00474806	9.93E-13	LMGP0301	PS(18:1_20:0)	PS(18:1(9Z)/20:0)
LMGP03010951	11.9934803	4.71E-15	0.00474806	9.93E-13	LMGP0301	PS(18:1_20:0)	PS(20:0/18:1(9Z))
LMGP03010497	10.9135798	1.36E-15	0.00416001	3.27E-13	LMGP0301	PS(19:0_19:1)	PS(19:1(9Z)/19:0)
LMGP03010467	10.9135797	1.36E-15	0.00416001	3.27E-13	LMGP0301	PS(19:0_19:1)	PS(19:0/19:1(9Z))
LMGP01030014	9.78589219	0	0	0	LMGP01030014	PC(P-18:0_22:6)	PC(P-18:0/22:6(4Z,7Z,10Z,13Z,16Z,19Z))
LMGP03010222	0.5917713	6.94E-33	1.22E-05	5.70E-28	LMGP0301	PS(16:1_22:0)	PS(16:1(9Z)/22:0)
LMGP03010731	0.59177126	6.94E-33	1.22E-05	5.70E-28	LMGP0301	PS(16:0_22:1)	PS(22:1(11Z)/16:0)
LMGP03010199	0.59177126	6.94E-33	1.22E-05	5.70E-28	LMGP0301	PS(16:0_22:1)	PS(16:0/22:1(11Z))
LMGP03010706	0.59177115	6.94E-33	1.22E-05	5.70E-28	LMGP0301	PS(16:1_22:0)	PS(22:0/16:1(9Z))
