# Supplementary material for: LipidFrag: Improving reliability of in silico fragmentation of lipids and application to the Caenorhabditis elegans lipidome
Source: PLoS One. 2017 Mar 9;12(3):e0172311. doi: 10.1371/journal.pone.0172311 (PMC5344313; doi:10.1371/journal.pone.0172311)
Supplement: S8 Table — (PDF) [file pone.0172311.s015.pdf]

S8 Table. *LipidFrag's improvement of ranks for training MS/MS spectra in positive ion mode.*

	FCP-Threshold	LMGL0301	LMGP0101	LMGP0201	LMGP0301	LMGP0601	LMSP0201 LMSP0202	All
Top 1	0	6	25	34	6	82	156	309
	0.1	6	25	34	6	82	156	309
	0.2	6	25	34	6	82	156	309
	0.3	6	25	34	6	82	156	309
	0.4	6	25	34	6	82	156	309
	0.5	6	25	34	6	82	155	308
	0.6	6	4	34	6	82	155	287
	0.7	6	0	34	6	82	154	282
	0.8	6	0	34	6	82	148	276
	0.9	6	0	34	6	82	124	252
Median Rank	0	3	2	2	2	1	1	1
	0.1	3	2	2	2	1	1	1
	0.2	3	2	2	2	1	1	1
	0.3	3	2	2	2	1	1	1
	0.4	3	2	2	2	1	1	1
	0.5	3	2	2	2	1	1	1
	0.6	3	2	2	2	1	1	1
	0.7	3	2	2	2	1	1	1
	0.8	3	2	2	2	1	1	1
	0.9	3	0	2	2	1	1	1
Mean Rank	0	3.08	5.83	1.73	1.88	1.00	1.17	2.09
	0.1	3.08	5.83	1.73	1.88	1.00	1.00	2.07
	0.2	3.08	5.83	1.73	1.88	1.00	1.00	2.07
	0.3	3.08	5.83	1.73	1.88	1.00	1.00	2.07
	0.4	3.08	5.83	1.73	1.88	1.00	1.00	2.07
	0.5	3.08	3.00	1.73	1.88	1.00	1.00	1.60
	0.6	3.08	1.69	1.73	1.88	1.00	1.00	1.41
	0.7	3.08	2.00	1.73	1.88	1.00	1.00	1.41
	0.8	3.08	2.00	1.73	1.88	1.00	1.00	1.42
	0.9	3.08	0.00	1.73	1.88	1.00	1.00	1.43
Number Spectra	0	25	71	88	51	82	172	489
	0.1	25	71	88	50	82	159	475
	0.2	25	71	88	50	82	159	475
	0.3	25	71	88	50	82	159	475
	0.4	25	71	88	50	82	159	475
	0.5	25	56	88	50	82	158	459

	0.6	*25*	*13*	*88*	*50*	*82*	*156*	*414*
	0.7	*25*	*9*	*88*	*49*	*82*	*155*	*408*
	0.8	*25*	*7*	*88*	*49*	*82*	*149*	*400*
	0.9	*25*	*0*	*88*	*49*	*82*	*125*	*369*
