# Supplementary material for: LipidFrag: Improving reliability of in silico fragmentation of lipids and application to the Caenorhabditis elegans lipidome
Source: PLoS One. 2017 Mar 9;12(3):e0172311. doi: 10.1371/journal.pone.0172311 (PMC5344313; doi:10.1371/journal.pone.0172311)
Supplement: S9 Table — (PDF) [file pone.0172311.s016.pdf]

S9 Table. *LipidFrag's improvement of ranks for training MS/MS spectra in negative ion mode.*

	FCP-Threshold	LMGP0601	LMSP0201 LMSP0202	LMGP0101 LMGP0201	LMGP0401	LMGP0301	All
Top 1	**0**	5	140	20	8	9	182
	**0.1**	5	129	20	9	9	172
	**0.2**	5	126	20	9	9	169
	**0.3**	4	125	20	9	9	167
	**0.4**	4	120	17	9	9	159
	**0.5**	3	119	14	9	9	154
	**0.6**	2	118	14	9	8	151
	**0.7**	2	117	14	9	8	150
	**0.8**	2	117	14	4	8	145
	**0.9**	1	105	14	3	8	131
Median Rank	**0**	2	1	2	2	2	2
	**0.1**	2	1	2	2	2	2
	**0.2**	2	1	2	2	2	2
	**0.3**	2	1	2	2	2	2
	**0.4**	2	1	2	2	2	2
	**0.5**	2	1	2	2	2	2
	**0.6**	2	1	2	2	2	2
	**0.7**	2	1	2	2	2	2
	**0.8**	2	1	2	2	2	2
	**0.9**	2	1	2	2	2	2
Mean Rank	**0**	2.55	1.28	2.23	1.83	1.75	1.83
	**0.1**	2.48	1.25	2.24	1.78	1.75	1.82
	**0.2**	2.42	1.26	2.24	1.78	1.77	1.82
	**0.3**	2.42	1.24	2.24	1.78	1.77	1.81
	**0.4**	2.42	1.24	2.27	1.78	1.77	1.82
	**0.5**	2.40	1.24	2.29	1.78	1.77	1.81
	**0.6**	2.39	1.24	2.29	1.78	1.77	1.81
	**0.7**	2.38	1.14	2.30	1.78	1.76	1.79
	**0.8**	2.34	1.06	2.30	1.89	1.75	1.78
	**0.9**	2.31	1.00	2.31	1.89	1.74	1.79
Number spectra	**0**	78	189	128	41	36	472
	**0.1**	75	170	125	41	36	447
	**0.2**	73	167	123	41	36	440
	**0.3**	71	164	121	41	36	433
	**0.4**	71	158	118	41	36	424
	**0.5**	68	157	113	41	36	415

	0.6	*62*	*155*	*112*	*41*	*35*	*405*
	0.7	*58*	*136*	*110*	*41*	*35*	*380*
	0.8	*56*	*125*	*105*	*35*	*35*	*356*
	0.9	*51*	*105*	*103*	*27*	*35*	*321*
